# Competing pathways in electrocarboxylation on copper electrodes: defining reactivity regimes through substrate activation and CO_2_ reduction

**DOI:** 10.1039/d6fd00073h

**Published:** 2026-10-09

**Authors:** Eleni Menou Aikateriniadou, Connor Deacon-Price, Nina Chen, Myrthe Zwart, Didjay F. Bruggeman, Amanda C. Garcia

**Affiliations:** a Van't Hoff Institute for Molecular Sciences, University of Amsterdam Science Park 904 1098 XH Amsterdam The Netherlands a.c.garcia@uva.nl didjaybruggeman@gmail.com

## Abstract

Electrocarboxylation of organic substrates offers a promising route for CO_2_ utilization in sustainable synthesis, but its practical application is limited by poor selectivity arising from competing electrochemical pathways. Here, we investigate the electrocarboxylation of benzyl bromide, benzaldehyde, and styrene on polycrystalline copper electrodes in acetonitrile to elucidate how substrate structure and applied potential govern reaction selectivity. By combining cyclic voltammetry, bulk electrolysis, and *in situ* FTIR spectroscopy, we found electrocarboxylation does not follow a single mechanistic pathway but is instead controlled by the competition between substrate activation, CO_2_ reduction, and parallel side reactions. Benzyl bromide exhibits the highest electrocarboxylation efficiency, consistent with facile substrate activation and rapid coupling with CO_2_. In contrast, benzaldehyde forms a ketyl intermediate that predominantly undergoes dimerization and hydrogenation, limiting carboxylation. For styrene, the more negative potentials required for substrate activation overlap with CO_2_ reduction, resulting in a regime dominated by CO_2_-derived products and minimal electrocarboxylation. These findings demonstrate that the relative alignment of substrate and CO_2_ reduction potentials defines distinct reactivity regimes that determine electrocarboxylation selectivity. This mechanistic framework contributes to the basis for rational design of electrochemical systems for efficient CO_2_ utilization.

## Abstract 2

Electrocarboxylation on Cu is governed by the balance between substrate activation and competing CO_2_ reduction pathways.

## Graphical abstract

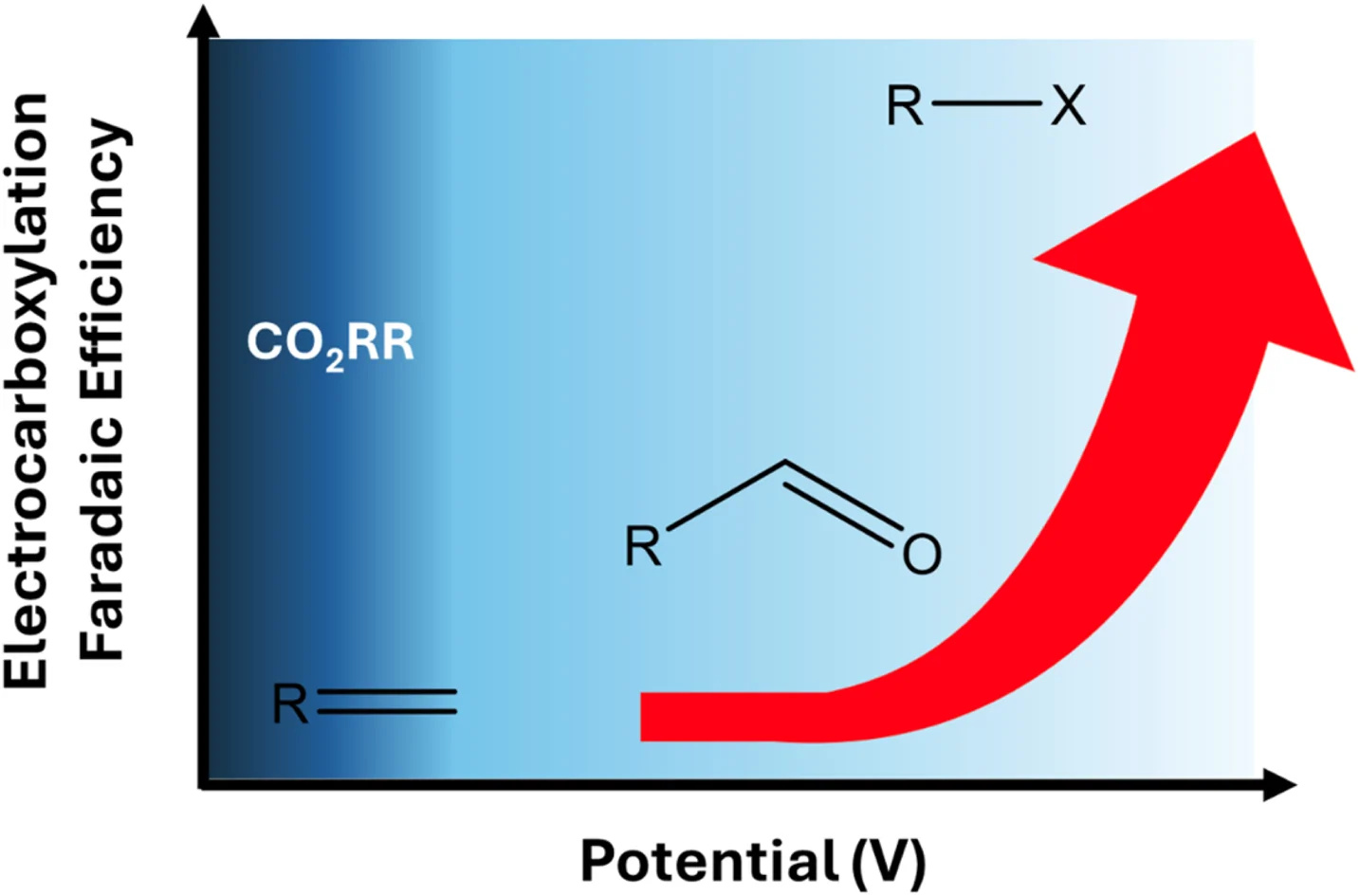

## 1. Introduction

The increasing concentration of carbon dioxide (CO_2_), mainly associated with the extensive use of fossil fuels, has motivated the development of strategies that use CO_2_ as a carbon source in chemical synthesis.^1^ Among these strategies, CO_2_ fixation into reactive organic substrates is attractive because it enables the formation of carboxylic acids, carbamates, and carbonates under relatively mild conditions.^2,3^

Carboxylic acids are particularly relevant due to their broad use in polymers, pharmaceuticals, and fine chemicals. Therefore, methods that reduce the dependence on fossil-derived feedstocks for carboxylic acid synthesis are important for the development of more sustainable chemical processes.^2–4^

Electrocarboxylation is a promising route to carboxylic acids because it uses electrons as reductants and can be powered by renewable electricity. The process enables direct incorporation of CO_2_ into organic molecules through electrochemically generated reactive intermediates, providing a route to carbon–carbon bond formation under relatively mild conditions. However, achieving high selectivity remains challenging because these intermediates can undergo multiple competing pathways. Alongside carboxylation, hydrogenation, dimerization, disproportionation, and further reduction may occur, while CO_2_ reduction can also compete for electrons at the cathode surface (Scheme 1).^3–7^

**Scheme 1:**
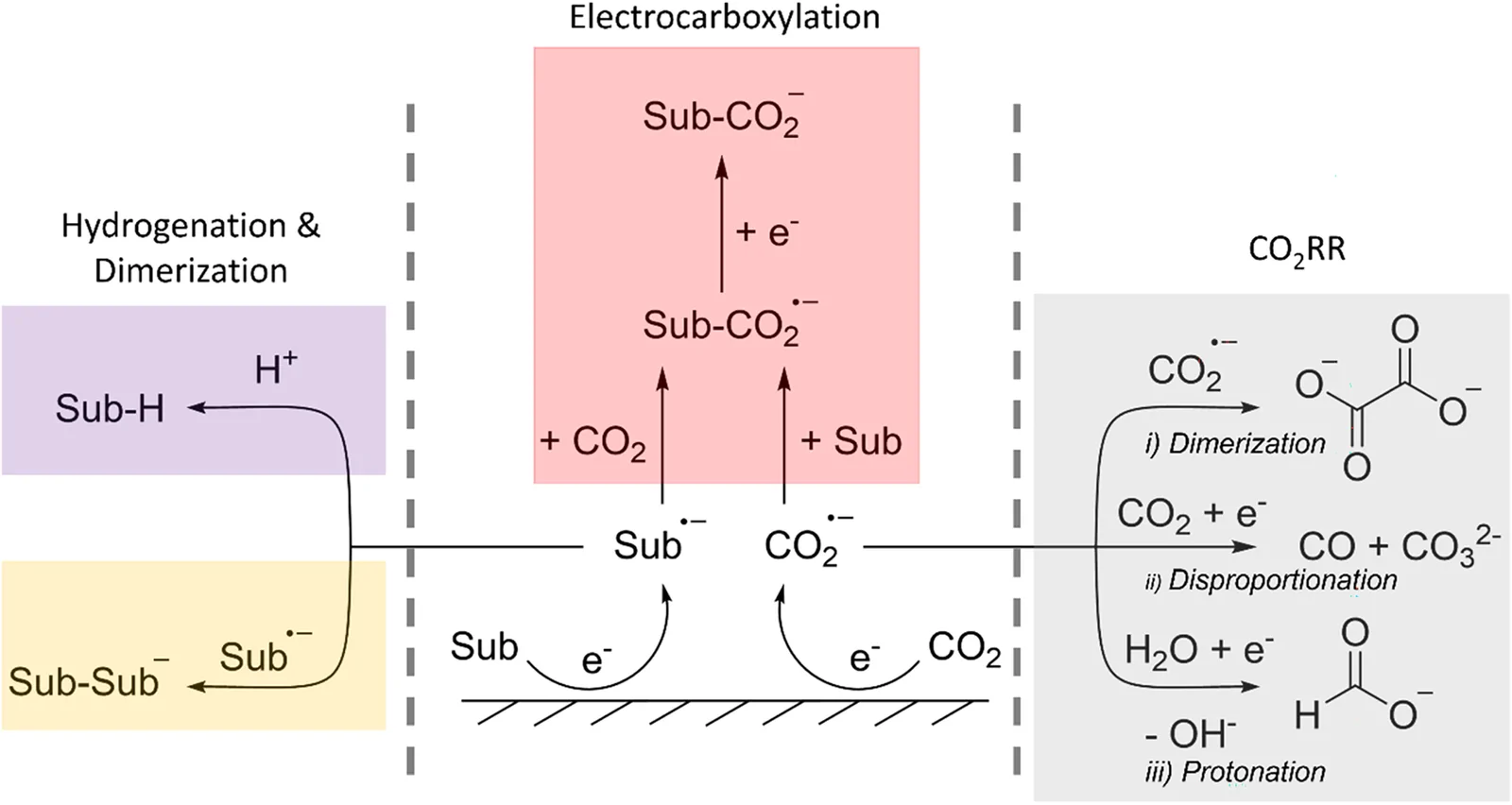
An overview of the parallel cathodic reactions that might occur during the electrocarboxylation reaction: molecular CO_2_RR (grey box), hydrogenation (purple box), dimerization (yellow box), and electrocarboxylation (red box).

Electrocarboxylation selectivity is therefore determined by how reactive intermediates are formed and consumed at the electrode interface. Depending on the substrate, electrochemical activation can generate radical or anionic species that may react with CO_2_, but can also undergo hydrogenation, dimerization, disproportionation, or further reduction. The fate of these intermediates depends on the substrate structure, solvent, electrolyte, proton availability, and electrode material, which together influence electron transfer, adsorption/desorption, and competition with CO_2_ reduction.^6–8^

The reaction outcome can also be affected by the cell configuration and anodic process. Sacrificial anodes such as Mg, Zn, or Al are commonly used to facilitate reductive electrocarboxylation, but they introduce metal consumption, waste generation, and possible effects of dissolved metal ions on the cathodic reaction.^3–5,9,10^ Non-sacrificial anodes avoid these issues, but may require less favorable anodic reactions and higher cell potentials.^11^

Although electrocarboxylation is often discussed in terms of substrate-first or CO_2_-first activation pathways (Scheme 2), these are limiting descriptions of a broader reaction network. In practice, especially under aprotic conditions, selectivity is governed by the relative rates of substrate activation, CO_2_ reduction, and parallel reactions such as hydrogenation, dimerization, and electrolyte decomposition, rather than by a single elementary step.^9,10^

**Scheme 2:**
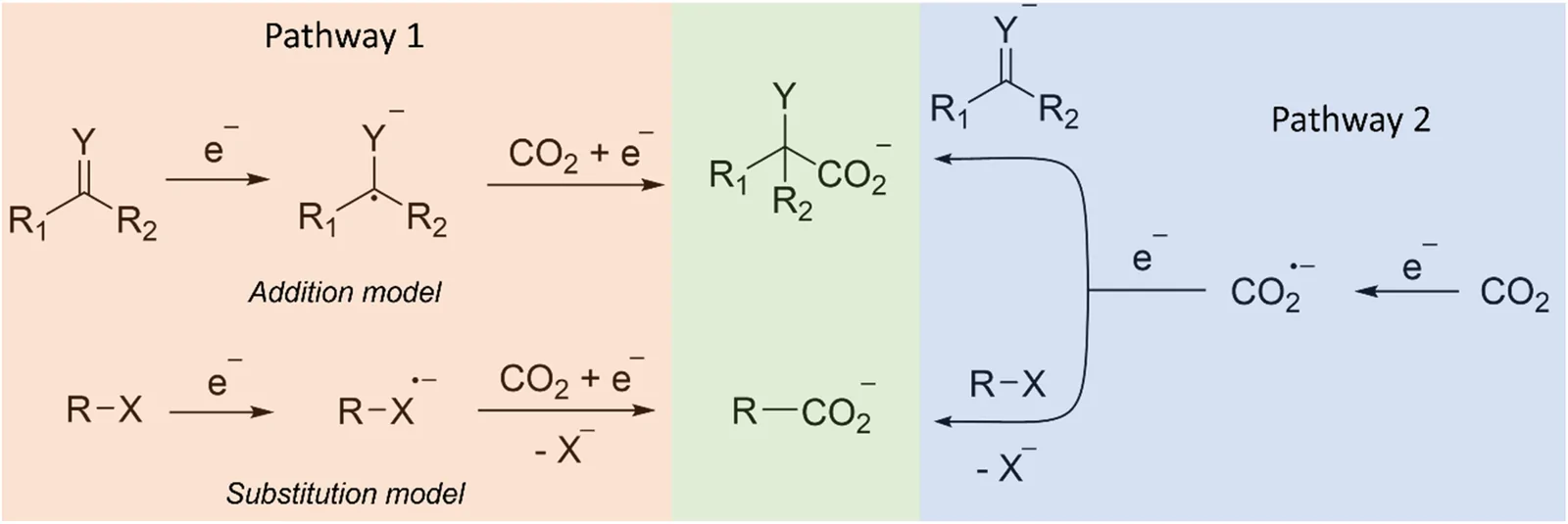
An overview of the possible reaction pathways in which electrocarboxylation can occur. Pathway 1 (orange area) proceeds by first activating the substrate *via* an electron transfer, or Pathway 2 (blue area) where CO_2_ is the site of initial electron transfer. Pathway 1 can occur *via* the addition model, or substitution model, depending on the functional group of the substrate. Both pathways lead to the same carboxylic acid products (green area). X = halide, SO_2_Ph, OH, OAc; Y = C, O, N, S.

Previous studies have reported electrocarboxylation of organic halides, aldehydes, and alkenes on several cathode materials, including Pb, Ag, carbon-based electrodes, and Cu-based systems.^12,13^ These studies have provided important insight into individual transformations and catalyst optimization.^15–29^ However, a direct comparison of different substrate classes under the same conditions remains less developed, particularly on catalytically active Cu electrodes where CO_2_ reduction can compete strongly with organic substrate activation.^14^

Our previous work on Pb cathodes showed that electrocarboxylation selectivity depends strongly on electrolysis conditions and substrate structure. Benzaldehyde and benzyl bromide were consistent with predominantly substrate-activated pathways, whereas styrene showed more complex behavior with competing mono- and dicarboxylation products.^12^ Because Pb is comparatively less active for direct CO_2_ reduction, these studies provided insight into substrate-controlled electrocarboxylation. In contrast, Cu can actively promote CO_2_ reduction, which may alter the balance between electrocarboxylation and competing CO_2_-derived pathways.^14,30–33^

Here, we investigate the electrocarboxylation of benzyl bromide, benzaldehyde, and styrene on polycrystalline Cu electrodes in acetonitrile solvent under identical conditions. By combining cyclic voltammetry, bulk electrolysis, and *in situ* FTIR spectroscopy, we examine how substrate activation, CO_2_ reduction, and side reactions compete at the Cu surface. Whereas benzyl bromide gives the highest electrocarboxylation efficiency, consistent with facile C–Br bond activation and rapid coupling with CO_2_, benzaldehyde forms a ketyl intermediate that mainly undergoes dimerization and hydrogenation, limiting CO_2_ incorporation. Styrene requires more negative potentials for activation, where CO_2_ reduction on Cu becomes strongly competitive. Together, these results define substrate-controlled, competitive, and CO_2_-dominated regimes for electrocarboxylation on Cu electrodes.

## 2. Materials and methods

### 2.1. Experimental conditions and reactants

Reagents and solvents were used without extra purification unless it is mentioned otherwise. Ultrapure water (Merck Milli-Q IQ 7000 Ultrapure Lab Water System, Millipak 0.22 µm Filter) with total organic carbon content <3 ppb and a resistivity higher than 18 M Ω cm at room temperature was used in all the experiments. Before each experiment, organic traces were removed by cleaning all glassware by soaking overnight with 0.1 M H_2_SO_4_ (95–98%, Sigma-Aldrich) in an aqueous solution of 0.0063 M KMnO_4_ (VWR International B.V., GPR RECTAPUR). Afterwards, traces of the MnO_4_^−^ was decomposed in dilute piranha solution (3 : 1 v/v H_2_SO_4_ (95–98%, Sigma-Aldrich)/H_2_O_2_ (30%, Sigma-Aldrich). The glassware was then rinsed several times in Milli-Q water, boiled three times and stored in the oven at 130 °C for at least 45 min to evaporate residual water. Solutions were purged for at least 15 minutes with argon gas (Ar, 99.999%, Linde) or carbon dioxide gas (CO_2_, 99.5%, Linde). All the reactions were run under atmospheric pressure and room temperature.

The studied substrates were benzyl bromide (BenzBr, 99%, Fischer Scientific), benzaldehyde (BenzAl, 99%, Sigma-Aldrich) and styrene (Str, 99.9% Sigma-Aldrich). 0.1 M tetrabutylammonium hexafluorophosphate (TBAPF_6_, Supelco) was used as the electrolyte, and acetonitrile (MeCN, 99.9% AcroSeal Fischer Scientific) was used as the solvent. We used an aprotic solvent to increase the organic molecules' solubility while preventing hydrogen evolution reaction and formation of more nucleophilic species. The electrolytic solution was further dried over 3 Å molecular sieves (Supelco) which were flame dried under vacuum on a Schlenk Line for at least 24 h. The average water content of the electrolyte was 50 ± 5 ppm, measuring a 0.3 mL sample on a Metrohm 831FK Coulometer. Molecular sieve dust was eliminated with a disc filter (0.22 µm PTFE membrane filter).

### 2.2. Electrochemical experiments

#### 2.2.1. Cyclic voltammetry

Cyclic voltammetry (CV) was conducted using a three-electrode setup in an undivided cell, connected to an Ivium Vertex.50V1A potentiostat. Polycrystalline Cu wire (MaTecK, 99.99% metal purity, ∅ = 0.1 cm, *L* = 3 cm) served as the working electrode, with graphite (MaTecK, ∅ = 0.6 cm) as the counter electrode, and a commercial leak-free Ag/Ag^+^ electrode (Alvatek) as the reference. Solution resistivity was measured by impedance spectroscopy before each experiment, and the potential was *iR* drop corrected by 85% of the solution resistance based on eqn (1):

1 *E*_corrected_ = *E* − [(*i* × *R*_u_) × 85%]

where *R*_u_ is uncompensated measured resistance.

Prior to use, the Cu electrode was electropolished in a 66% H_3_PO_4_ (Alfa Aesar, 85%) aqueous solution in a 2-electrode configuration, with a Cu plate as counter electrode. A voltage of 3.0 V was applied for 15 s before returning to 0.0 V for three cycles. Before each experiment, the Cu surface was characterized by running a “blank” cyclic voltammogram in Ar-deaerated 0.1 M NaOH(aq) (Fig. S1) with a scan rate of 50 mV s^−1^, in a one-compartment electrochemical cell configuration, using a glassy carbon (MaTecK) and a reversible hydrogen electrode (RHE) (Hydroflex) as counter and reference electrodes, respectively.

For the cyclic voltammetry experiments, 5 mL of the standard electrolyte (0.1 M TBAPF_6_ in MeCN) was added to a home-made three-compartment electrochemical cell and the solution was magnetically stirred during gas deaeration/saturation, and between each measurement. Control measurements of Ar-deaerated and saturated with CO_2_ gas were taken, after bubbling for a minimum of 15 min through a clean PTFE tube with a continuous flow of approximately 50 mL min^−1^. Following that, 50 mM of the substrate (benzaldehyde, styrene, or benzyl bromide) was added into the cell, which was gassed with saturated CO_2_ gas and degassed with Ar. During the measurements, gas flow was kept above the electrolyte (head space) to avoid oxygen dissolving into the solution. The cyclic voltammograms were recorded with a scan rate of 50 mV s^−1^ from −0.5 V to −2.5 V *vs.* Ag/Ag^+^ (benzaldehyde and benzyl bromide) and from −0.5 V to −3.0 V *vs.* Ag/Ag^+^ (styrene) and a total of three scans were taken.

#### 2.2.2. Bulk electrolysis – chronoamperometry

Bulk electrolysis was performed in a PEEK two-compartment (divided) cell designed for on-line gas chromatography (GC) analysis during the experiment (the cell design is shown in the SI, Fig. S1C). The cell was operated by an IVIUM Vertex.s potentiostat/galvanostat controlled by IviumSoft 4.1113 software. In potentiostatic mode the potential was corrected for the *iR* drop by applying a correction of 85% of the resistance, determined at the start of the experiment by electrochemical impedance spectroscopy (EIS), the default setting in IviumSoft. The anodic (counter electrode compartment, CEC) and cathodic (working electrode compartment, WEC) compartments were separated by a pre-treated Nafion-117 membrane that was patted dry with tissue paper. The membrane was soaked in acetonitrile (MeCN) for 30 min before being installed between the two compartments to remove the residual water. A Cu wire (MaTecK, 99.99% metal purity), and miniature leakless Ag/Ag^+^ (Alvatek) were used as working and reference electrodes, respectively. Although the supporting electrolyte was dried before use, the water content was not measured after cell assembly or after electrolysis. However, our previous work indicated that the water content increases from around 15 ppm to 100 ppm.^6^ Therefore, we cannot exclude that residual water associated with the Nafion membrane, electrolyte, or cell components contributes to the local proton availability during bulk electrolysis. This should be considered when interpreting hydrogenation products and ketyl-protonation pathways under these nominally aprotic conditions.

A solution of 8 mL of 0.1 M TBAPF_6_ in MeCN with or without 50 mM substrate was added to the cathodic compartment, while the anodic compartment consisted of a flame annealed Pt mesh as counter electrode and 8 mL of 0.1 M TBAPF_6_ in MeCN electrolyte. Ar or CO_2_ gas flow (20 mL min^−1^) was controlled using a Brooks Mass Flow Controller (Model O254) and the cell was checked for possible leakage before each experiment. A sample was injected over the online GC without applied potentials until the absence of atmospheric Ar and O_2_ was confirmed. The half-wave potentials were defined from the cyclic voltammograms as the middle potential of the reduction wave and chosen as the constant applied potentials for the bulk electrolysis experiments. This constant potential was applied for 2.5 h and the expected products were separated in gas and liquid phase. The resulting chronoamperometry plots were used to determine time-resolved current densities and passed charge (Fig. S2).

The quantitative analysis of the gas products detected in the gas chromatograms was performed using a GC (Agilent 990 Micro GC), with a split channel feed into two thermal conductivity detectors (TCD). A gas sample was taken and analysed every 10 min and analysed in two columns to determine products with different sizes. The first thermal conductivity detector (TCD) was a Molsieve column (MS5A SS 10 m × 0.25 mm × 30 µm BF, Agilent) to identify H_2_, O_2_, N_2_, CH_4_, CO with Ar as carrier gas (Nippon gases), injection temperature 110 °C, injection time 50 ms, backflush time 14 s (CP-Porabond column Q 3 m × 0.25 mm × 3 µm, Agilent), column temperature 80 °C, static pressure mode, initial pressure 200 kPa, run time 120 s. The second column used is a CPPoraPLOT U column (Poraplot U FS 10 m × 0.25 mm × 8 µm BF, Agilent) to identify ethene, ethane, propane, CO_2_ and H_2_O with He as carrier gas (Nippon gases). The injection temperature was 110 °C, injection time 50 ms, backflush time 0 s (CP-Porabond column Q 3 m × 0.25 mm × 3 µm, Agilent), column temperature 80 °C, static pressure mode, initial pressure 200 kPa and run time 120 s.

Liquid product analysis was performed with high performance liquid chromatography (HPLC, Agilent 1260, Infinity II) using an Aminex HPX-87H ion exclusion column (Bio-Rad), a refractive index detector (RID, G7162A) and a variable wavelength detector (VWD, G7114A). Six samples in triplicate (20 µL each) before the bulk electrolysis, and directly after filled to 1 mL total volume with ultrapure water. We used two columns to determine products with different polarity, thus the samples were filled with different solvents. For analysis of less polar and larger compounds produced from the substrate reactions, we dilute the samples with acetonitrile (980 µL, HPLC grade, Biosolve) and run them on a reversed-phase column (InfinityLab Poroshell 120 EC-C18). Poroshell samples were run at 30 °C column temperature using a solvent gradient which consisted at the start of the cycle of 90 : 10 5 mM H_2_SO_4_ (HPLC grade, 98%, Millipore; in ultrapure water) to acetonitrile (HPLC grade, Biosolve) which was reversed to 0 : 100 over 25 min with a flow rate of 0.5 mL min^−1^, injection volume of 5 µL and VWD set to 200 nm. To analyse small, polar compounds produced from CO_2_RR (formic acid, glycolic acid, oxalic acid), we filled the samples with Milli-Q (980 µL) and ran them on an ion exclusion column (Bio-Rad Aminex HPX-87H). Aminex samples were run at 50 °C column temperature using an aqueous mobile phase of 5 mM H_2_SO_4_ (HPLC grade, 98%, Millipore) solution in Milli-Q and every analysis cycle was performed for 35 min with a flow rate of 0.5 mL min^−1^ and injection volume of 20 µL.

### 2.3. *In situ* Fourier transform infrared (FTIR) spectroscopy

To investigate possible surface-adsorbed intermediates and products during the electrochemical experiments we performed *in situ* Fourier transform infrared (FTIR) spectroscopy. The spectra were measured on a Nicolet iS50 FTIR spectrometer connected with a liquid nitrogen cooled MCT detector. A Veemax III (PIKE Technologies) was housed on the spectrometer, and a home-made three-electrode spectro-electrochemical cell with a CaF_2_ prism at the cell's base was attached. 10 mL of 0.1 M TBAPF_6_ in MeCN electrolyte was added to the cell in addition to the relevant substrate. Pt wire and Ag/Ag^+^ (3.0 M KCl, ET069 leakless, ET06) were used as counter and reference electrodes, respectively. A polycrystalline Cu disk (CuPoly–*φ* 5 mm, Pine Research) was used as the WE, polished following the aforementioned procedure, which was placed vertically to the prism to build the desired thin layer configuration which was refreshed after a full experimental set. The FTIR spectra were performed in the range of 4000 to 1000 cm^−1^ and were recorded at potentials varying from −0.5 V to −2.5 V *vs.* Ag/Ag^+^ (benzaldehyde and benzyl bromide) or from −0.5 V to −3.0 V *vs.* Ag/Ag^+^ (styrene) under Ar or CO_2_ atmospheres. Spectra are presented as reflectance, based on eqn (2):

2 *A* = −log(*R*/*R*_0_)

where *R* is the single beam spectra obtained at the sample potential and *R*_0_ is the single beam spectra obtained at the reference potential. Positive bands (pointing up) in the spectra indicate species that are produced on or near the electrode surface at the sample potential. Negative bands (pointing down) in the spectra correspond to species that are present on or near the electrode surface and consumed at the sample potential. It is important to note that the thin layer configuration of FTIR experiments results in low currents and suppresses any possible Pt dissolution in the MeCN electrolyte.

## 3. Results and discussion

The electrocarboxylation behavior of benzyl bromide (BenzBr), benzaldehyde (BenzAl), and styrene (Str) on polycrystalline copper electrodes was investigated using cyclic voltammetry, bulk electrolysis, and *in situ* FTIR spectroscopy. Because cyclic voltammetry alone cannot unambiguously resolve coupled electrochemical–chemical mechanisms, the data are interpreted in combination with product analysis and spectroscopic observations to propose mechanistic trends.

### 3.1. Cyclic voltammetry

The cyclic voltammograms (CVs) of the different substrates (Fig. 1) were recorded in MeCN using a polycrystalline Cu working electrode (Cu_poly_) to probe their electrochemical behavior under Ar-deaerated and CO_2_-saturated conditions. The onset potentials and current responses provide an indication of the potentials at which substrate and/or CO_2_ reduction processes begin, allowing a qualitative comparison of their relative activation. Because CV alone cannot unambiguously resolve coupled electrochemical–chemical steps, the voltammetric data are used here mainly to identify potential regions where substrate reduction and CO_2_ reduction may compete. The mechanistic interpretation is further supported by the bulk electrolysis and *in situ* FTIR results discussed below. The onset and intensity of this response vary slightly between measurements, but in all cases CO_2_ reduction occurs within the potential window relevant for the reduction of the organic substrates. This indicates that substrate activation and CO_2_ reduction can overlap under the electrolysis conditions.

**Fig. 1:**
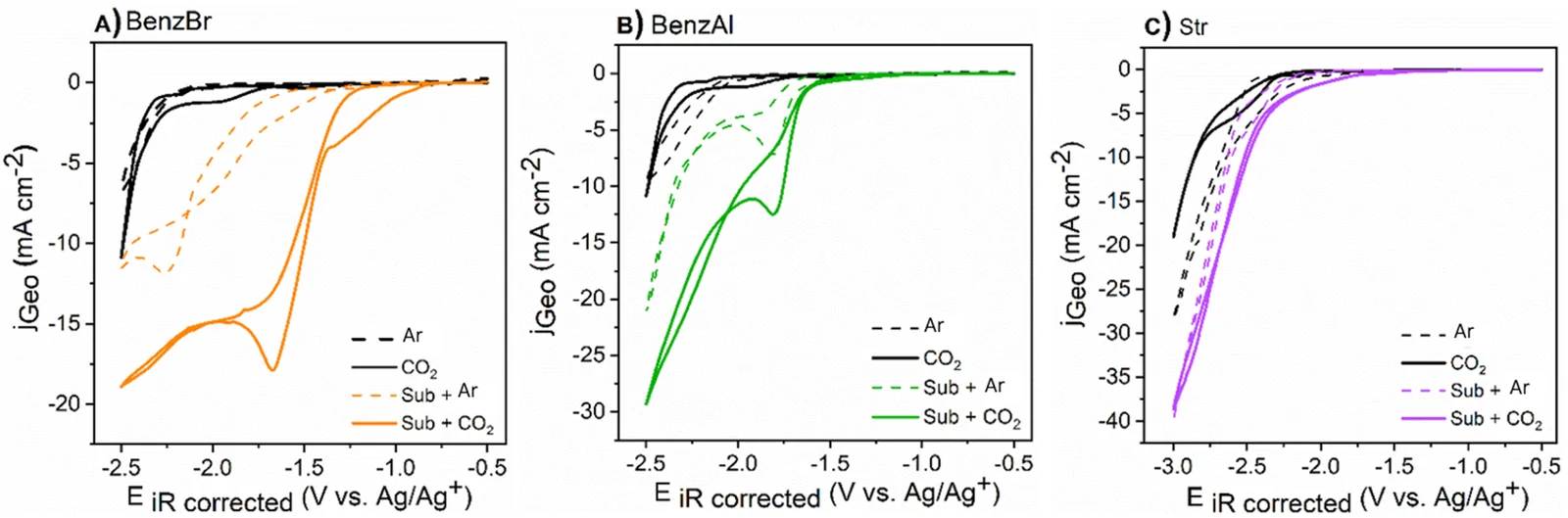
Cyclic voltammograms with and without 50 mM substrate with (A) benzyl bromide, (B) benzaldehyde and (C) styrene in 0.1 M TBAPF_6_ solution in MeCN (5 mL) with WE = Cu_poly_, CE = graphite, RE = Ag/Ag^+^. Solutions were Ar-deaerated (dashed line) and CO_2_-saturated (solid line). Scan rate = 50 mV s^−1^.

In the absence of substrate, CVs of Cu_poly_ under Ar (black dotted line) show negligible current until approximately −2.2 V, beyond which a sharp increase in cathodic current is observed, attributed to electrolyte decomposition.^35^ In CO_2_-saturated solution (black line), an additional cathodic peak is observed consistent with CO_2_ reduction on Cu in MeCN.^7^ These features indicate that CO_2_ can be electrochemically activated within the potential window relevant for substrate reduction, suggesting that both processes may compete under reaction conditions.

The CV profile of benzyl bromide in Ar-deaerated solution (Fig. 1A, orange dotted line) shows an irreversible reduction wave at approximately −2.4 V, which is consistent with a one-electron reduction process involving cleavage of the C–Br bond, generating a benzyl radical intermediate and bromide, as commonly proposed for organic halide electroreduction. Upon saturation with CO_2_ (Fig. 1A, orange solid line), the voltammetric response changes significantly, with the appearance of a cathodic feature at less negative potentials, around −1.4 to −1.6 V, together with an increase in current density. This positive shift suggests that the presence of CO_2_ facilitates a follow-up reaction after initial substrate activation, possibly by trapping the benzyl-derived intermediate and shifting the reaction equilibrium toward product formation.^34–37^

A second cathodic feature is observed at more negative potentials, in the region where CO_2_ reduction also occurs on the Cu electrode. This indicates that CO_2_ activation may occur concurrently under these conditions, giving rise to a regime in which substrate-derived radicals and CO_2_-derived intermediates can both be present. Therefore, although the first response is consistent with substrate activation, the CV also indicates that CO_2_ reduction can become competitive at more negative potentials.^14,38^ Therefore, the voltammetric behavior supports a substrate-initiated electrocarboxylation pathway for benzyl bromide, but also shows that more than one cathodic process may contribute under CO_2_-saturated conditions.

In the case of benzaldehyde (Fig. 1B), the CV profile in Ar-deaerated solution (green dotted line) shows two irreversible reduction waves, with onset potentials at approximately −1.5 V and −2.0 V. These features are consistent with sequential electron transfer processes, where the first reduction step is assigned to the formation of the ketyl radical anion (BenzAl˙^−^). This intermediate may subsequently undergo further reduction or follow-up reactions such as dimerization or protonation.^38^ Upon saturation with CO_2_ (green solid line), the overall voltammetric response changes only modestly compared to benzyl bromide. The onset of the first reduction wave remains largely unchanged. This behavior suggests that the initial formation of the ketyl intermediate is not strongly altered by the presence of CO_2_. Instead, CO_2_ appears to influence where follow-up reactions of the ketyl intermediate and CO_2_ reduction may both contribute. The CV data are therefore consistent with initial substrate reduction, but they also indicate that CO_2_ incorporation competes with other pathways rather than becoming the dominant process at this stage.^39^

In contrast to benzyl bromide and benzaldehyde, styrene reduction occurs at significantly more negative potentials. In Ar-deaerated solution (Fig. 1C, purple dotted line), a cathodic wave appears around −2.1 V, consistent with reduction of the alkene moiety. Upon saturation with CO_2_ (purple solid line), only a modest shift toward less negative potentials and an increase in current density are observed. Importantly, styrene activation begins in a potential region where CO_2_ reduction on Cu also becomes significant. Thus, the data do not indicate a simple sequence in which CO_2_ is necessarily reduced before styrene. Rather, they show that the potentials required for styrene activation strongly overlap with those where Cu promotes CO_2_ reduction.

This behavior differs from our previous observations on Pb electrodes,^12^ where styrene electrocarboxylation remained accessible under different electrolysis conditions. On Cu, the catalytic activity of the surface toward CO_2_ reduction leads to stronger competition between substrate reduction and CO_2_RR at highly cathodic potentials. This overlap is also consistent with previous reports showing that styrene electrocarboxylation is highly sensitive to the electrode material, proton availability, and applied conditions, with product distributions varying between hydrocarboxylation and dicarboxylation pathways.^40–42^

Overall, the CV results reveal three different electrochemical regimes. Benzyl bromide shows a pronounced positive shift in the presence of CO_2_, consistent with facile substrate activation followed by reaction with CO_2_. Benzaldehyde is reduced at relatively mild potentials, but the limited change upon CO_2_ addition suggests that ketyl follow-up chemistry remains important. Styrene requires more negative potentials for activation, placing substrate reduction in a region where CO_2_ reduction on Cu becomes strongly competitive. These trends provide the electrochemical basis for the product distributions observed during bulk electrolysis.

### 3.2. Bulk electrolysis

Bulk electrolysis experiments were performed to correlate the electrochemical behavior observed in cyclic voltammetry with product distribution and reaction selectivity. Chronoamperometry was conducted under both Ar-deaerated (Fig. S3) and CO_2_-saturated conditions (Fig. 2) at constant applied potentials selected from the half-wave potentials (*E*½) of the first reduction wave observed in the CO_2_-saturated voltammograms (Fig. 1). For clarity, the observed products are grouped into four categories: electrocarboxylation, hydrogenation, dimerization, and CO_2_ reduction products (Scheme 1). Fig. 2 summarizes the faradaic efficiencies of the liquid and gas products, while the corresponding partial currents are shown in Fig. S4. Substrate conversion, product yields, and product selectivities toward carboxylated product are summarized in Fig. S5. These metrics are consistent with the trends observed from the faradaic efficiencies shown in Fig. 2. Benzyl bromide exhibits high conversion under both Ar and CO_2_ atmospheres (>90%), but the product distribution changes in the presence of CO_2_, where phenylacetic acid becomes the major product and accounts for approximately 60% of the detected liquid products (Fig. S5G). In contrast, benzaldehyde shows a substantially lower conversion in CO_2_-saturated electrolyte compared to Ar (Fig. S5B), while the product distribution remains dominated by hydrobenzoin (Fig. S5H) isomers, indicating that dimerization continues to outcompete electrocarboxylation. Styrene displays only moderate conversion under both conditions (Fig. S5C) and produces only minor amounts of electrocarboxylation products, despite measurable substrate consumption. Instead, the product distribution under CO_2_ is largely associated with CO_2_ reduction products, consistent with the overlap between substrate activation and CO_2_ reduction observed in the cyclic voltammograms. Overall, the conversion and selectivity data support the existence of three distinct reactivity regimes on the Cu electrode: a substrate-controlled regime for benzyl bromide, a competitive regime for benzaldehyde, and a CO_2_RR-dominated regime for styrene.

**Fig. 2:**
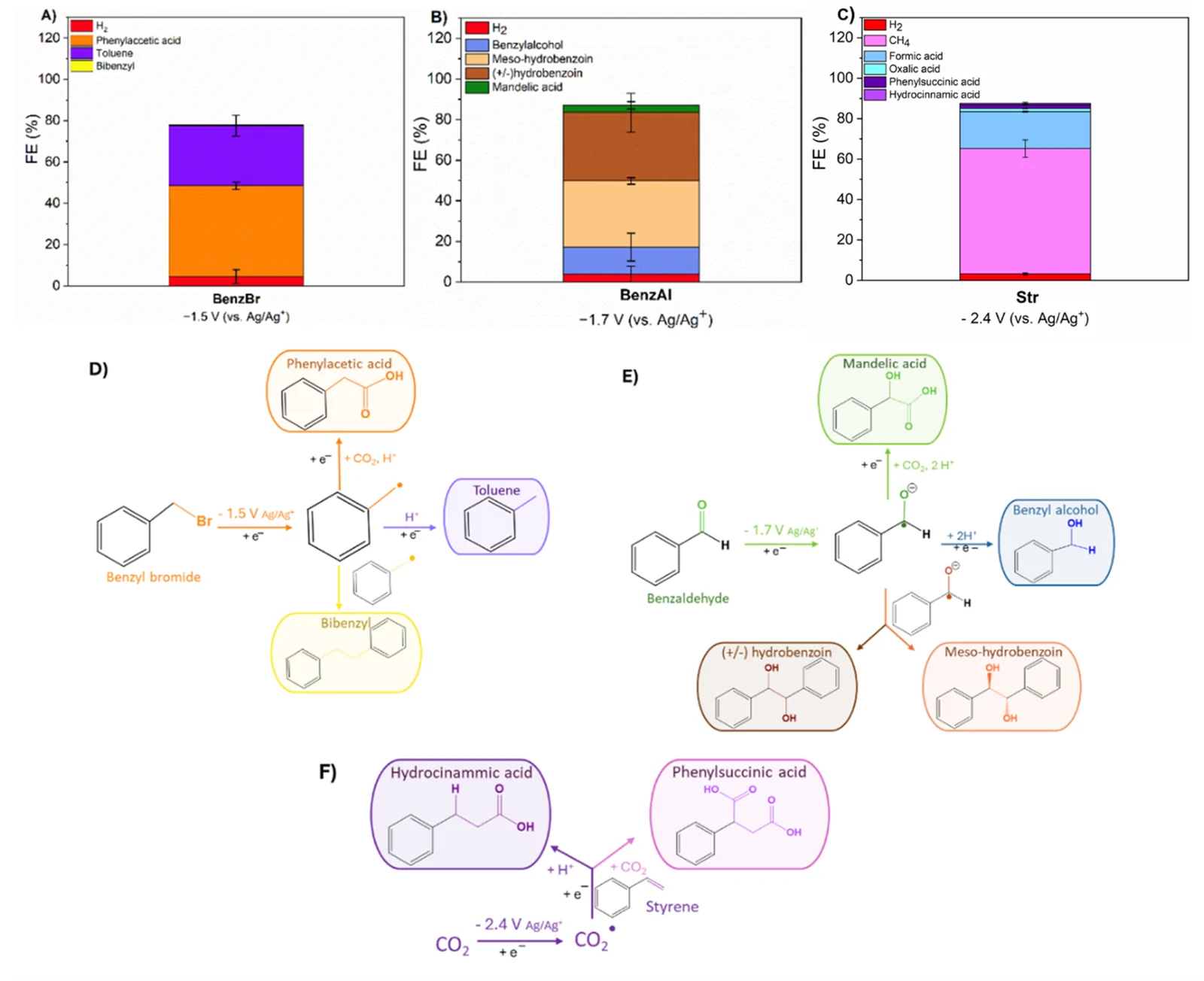
Faradaic efficiencies of the liquid and gas products, quantified by HPLC and online GC using a Cu_poly_ electrode, of 50 mM substrate; (A) benzyl bromide, (B) benzaldehyde or (C) styrene in 0.1 M TBAPF_6_ in CO_2_-saturated MeCN. Proposed reaction mechanisms of the electrocarboxylation of (D) benzyl bromide, (E) benzaldehyde and (F) styrene.

Bulk electrolysis of benzyl bromide was performed at −1.5 V *vs.* Ag/Ag^+^. Under Ar-deaerated conditions (Fig. S3A), the main products detected were H_2_ (18% FE), attributed to the reduction of residual water,^40,41^ and toluene (14% FE), formed by hydrogenolysis of the substrate.^43^ The remaining charge likely arises from undetected products and electrolyte decomposition.^44^

Under CO_2_-saturated conditions (Fig. 2A), phenylacetic acid becomes the dominant product, with a faradaic efficiency of 44%. Toluene is also formed in significant amounts (29% FE), whereas bibenzyl is detected only in minor quantities (0.3% FE). A small contribution from H_2_ evolution is also observed (4% FE). The formation of phenylacetic acid as the main product indicates that electrocarboxylation is favored for benzyl bromide under these conditions. At the same time, the formation of toluene shows that hydrogenolysis remains an important competing pathway.

This product distribution is consistent with a substrate-activated pathway in which reductive C–Br bond cleavage generates a benzyl-derived intermediate that can be trapped by CO_2_ to form phenylacetic acid.^35–43^ The low amount of bibenzyl suggests that radical–radical coupling is largely suppressed in the presence of CO_2_, allowing CO_2_ trapping to compete efficiently with dimerization. Similar substrate-controlled behavior was previously observed on Pb cathodes, where benzyl bromide electrocarboxylation was also dominated by substrate activation pathways. In addition, mechanistic studies on organic halide electrocarboxylation have shown that hydrogenolysis can compete with carboxylation even in aprotic solvents, because solvent- or electrolyte-derived proton transfer can contribute to the reduced side product. This provides a plausible explanation for the formation of toluene alongside phenylacetic acid in the present system. Overall, the relatively facile activation of benzyl bromide appears sufficient to maintain efficient electrocarboxylation on Cu, despite the catalytic activity of this surface toward CO_2_ reduction.

Bulk electrolysis of benzaldehyde was performed at −1.7 V *vs.* Ag/Ag^+^ (Fig. 2B). Under Ar-deaerated conditions (Fig. S3B), the dominant products are the hydrobenzoin isomers, indicating that dimerization is the preferred pathway following benzaldehyde reduction. This behavior is consistent with the formation of a ketyl radical intermediate, which readily undergoes radical–radical coupling. In CO_2_-saturated electrolyte, only a small amount of mandelic acid is detected (3.6% FE). Dimerization products remain dominant, with *meso*-hydrobenzoin and (±)-hydrobenzoin together accounting for approximately 67% FE. Benzyl alcohol is also formed with a faradaic efficiency of 13.4%, showing that hydrogenation pathways become significant in the presence of CO_2_.

These results indicate that, although benzaldehyde is reduced at relatively mild potentials, the resulting ketyl intermediate does not efficiently undergo CO_2_ incorporation. Instead, dimerization remains the main reaction pathway. The formation of benzyl alcohol also shows that protonation and further reduction pathways compete with carboxylation.^44,45^ Similar behavior was previously observed on Pb cathodes, where ketyl radical dimerization also dominated over electrocarboxylation. Compared with Pb,^12^ however, Cu gives a larger contribution from hydrogenation products, suggesting that the catalytic surface and local electrolyte environment influence the fate of the ketyl intermediate under CO_2_-saturated conditions. Taken together, the benzaldehyde results are consistent with a substrate-initiated reaction network in which ketyl radical formation is followed by competing dimerization, hydrogenation, and carboxylation pathways. Under the present conditions, dimerization is the dominant channel, limiting efficient CO_2_ incorporation.

Bulk electrolysis of styrene was performed at −2.4 V *vs.* Ag/Ag^+^. Under Ar-deaerated conditions (Fig. S3C), H_2_ was the main detected gaseous product, while HPLC analysis indicated partial styrene consumption, suggesting the formation of undetected side products, such as oligomeric or polymeric species. In CO_2_-saturated electrolyte (Fig. 2C), the dominant products arise from CO_2_ reduction, including formic acid and methane. Only minor amounts of the electrocarboxylation products hydrocinnamic acid and phenylsuccinic acid are detected. These observations indicate that styrene activation is possible on Cu, but that CO_2_ reduction becomes strongly competitive at the highly cathodic potentials required for substrate conversion.

This interpretation agrees with the cyclic voltammetry results, where styrene reduction occurs in a potential region that strongly overlaps with CO_2_ reduction on Cu. Therefore, the low electrocarboxylation efficiency does not indicate that styrene is intrinsically unsuitable for electrocarboxylation, but rather that under the present conditions CO_2_RR dominates the reaction environment. This conclusion is consistent with previous styrene electrocarboxylation studies, which showed that product selectivity is highly sensitive to proton availability, electrode material, and electrolysis conditions. In optimized systems, β-hydrocarboxylation can be favored when proton transfer is controlled, whereas dicarboxylation is promoted when the carboxylated intermediate undergoes further reduction and reacts with a second CO_2_ molecule. More recent continuous-flow and photoelectrochemical studies further show that styrene carboxylation can be improved by controlling residence time, electron flux, and interfacial charge transfer.^40–42^

Overall, the bulk electrolysis results show that the three substrates occupy distinct reactivity regimes on the Cu electrode. Benzyl bromide gives the highest electrocarboxylation efficiency because substrate activation is facile and CO_2_ trapping efficiently competes with dimerization, corresponding to a substrate-controlled regime. Benzaldehyde is also activated at relatively mild potentials, but the resulting ketyl intermediate preferentially undergoes dimerization and hydrogenation, placing the reaction in a competitive regime where CO_2_ incorporation remains limited. In contrast, styrene requires substantially more negative potentials for activation, placing substrate conversion in a potential region where CO_2_ reduction on Cu becomes increasingly competitive. Consequently, only minor amounts of carboxylation products are formed despite measurable substrate consumption, corresponding to a CO_2_RR-dominated regime.

Comparison with our previous studies on Pb cathodes highlights the role of electrode material in controlling this balance. While benzyl bromide and benzaldehyde show similar substrate-controlled behavior on both cathodes, styrene exhibits a much stronger dependence on the electrode material. On Pb, styrene electrocarboxylation remained accessible under different electrolysis conditions, whereas on Cu the higher activity toward CO_2_ reduction shifts the reaction environment toward CO_2_RR at the potentials required for substrate activation. These observations indicate that electrocarboxylation selectivity is governed not only by the ease of substrate activation, but also by the fate of the resulting intermediates and the extent to which CO_2_ reduction overlaps with substrate conversion.

### 3.3. Electrochemical *in situ* FTIR

To better understand the differences involved in the mechanism of the reaction for each of the three substrates studied, we performed *in situ* FTIR analysis, which allows us to identify possible products and intermediates of the reaction. Fig. 3 compares the spectra obtained for Cu_poly_ under Ar-deaerated and CO_2_-saturated conditions, without the presence of the substrate. Full assignment tables are available (Tables S1 and S2).

**Fig. 3:**
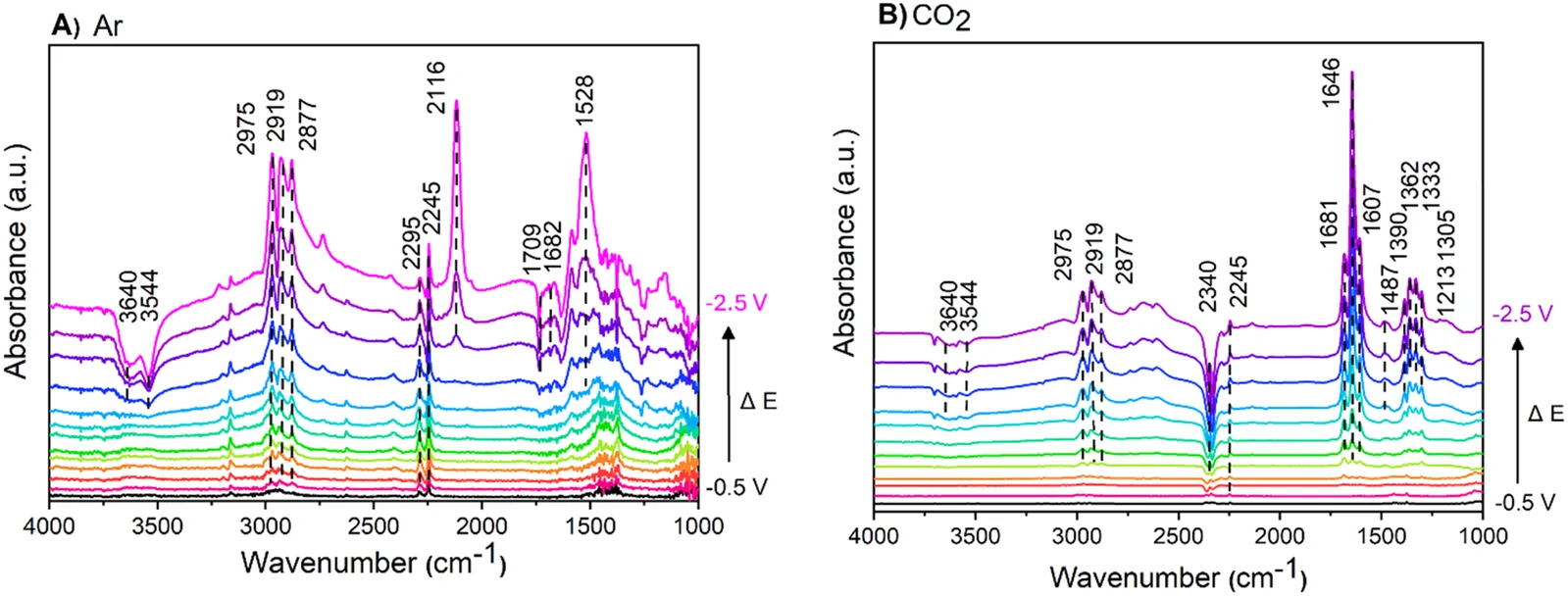
Electrochemical *in situ* FTIR spectra performed in pure MeCN electrolytic solution under Ar-deaerated (A) and CO_2_-saturated (B) conditions on a Cu_poly_ cathode in 0.1 M TBAPF_6_. Background spectra were taken at −0.5 V. Spectra in (A) are recorded at −0.5 V, then −1.0 V, increasing by −200 mV until −1.8 V, then increasing by −100 mV until −2.5 V. Spectra in (B) are recorded at −0.5 V, then −1.0 V, increasing by −400 mV until −1.4 V, increasing by 100 mV until −2.1 V, then increasing by −200 mV until −2.5 V.

At more negative potentials, the spectra in Fig. 3 show negative bands at 3640 and 3544 cm^−1^, which are assigned to the O–H stretching mode of residual water, indicating its consumption.^31^ These occur at more negative wavenumbers than in aqueous solvent owing to the difference in hydrogen-bonding networks observed in MeCN.^41^ These bands are specifically caused by free dangling O–H groups, which are present in hydrophobic hydration shells generated by the cation.^46–48^ Similar to our previous work, these bands are suppressed in CO_2_-saturated solution (Fig. 3B), suggesting that the presence of CO_2_ changes the interfacial proton/water environment and suppresses HER under these conditions.^7^

In addition, positive bands at 2975, 2919, and 2877 cm^−1^ (Fig. 3A) correspond to the C–H stretching of the TEA^+^ cation.^31^ The increase in intensity of these bands as a function of applied potential is due to an accumulation of cations at the electrode–electrolyte interface,^31^ and within the thin layer between prism and electrode.^31,49^ As the region probed by the IR beam remains constant throughout the experiment, more cations are detected relative to the background. This effect is present throughout all the FTIR spectra. Bands at 2295 and 2245 cm^−1^, especially clear in Ar-deaerated conditions, are assigned to C–N stretching of MeCN. A negative band at 1709 cm^−1^ appears alongside water consumption, indicated by the O–H stretching bands. We therefore assign this feature to a slightly red-shifted O–H bending mode of H_2_O, which can occur due to restricted hydrogen bonding in MeCN.^7,35^ The positive band at 1682 cm^−1^ is assigned to acetamide formation, while the bands at 2116 and 1528 cm^−1^ are attributed to 3-aminocrotononitrile, a dimerization product formed under very dry conditions.^36^

When the electrolyte is saturated with CO_2,_ the bands related to MeCN degradation are strongly suppressed, while the positive bands at 1681, 1646, 1607, 1390, 1362, 1333, 1305, and 1213 cm^−1^ are observed. These bands have previously been assigned to bicarbonate and carbonate species.^31^ Based on our previous work, the band at 1213 cm^−1^ may also contain a contribution from the C–O stretching vibration of oxalate, particularly under dry conditions.^7^ These positive bands appear together with a negative band at 2340 cm^−1^, assigned to the C=O stretching mode of dissolved CO_2_, indicating CO_2_ consumption near the electrode surface.

With the identification of the background electrolytic activity and CO_2_ reduction products, we can now more accurately investigate the spectral bands corresponding to substrate reduction processes, such as hydrogenation, dimerization, and electrocarboxylation. For clarity, we compare the spectra obtained in Ar-deaerated and CO_2_-saturated electrolytes for each substrate at their respective half-wave potentials, highlighting the bands associated with substrate reduction (Fig. 4). Additionally, we provide full potential-resolved spectra for each substrate along with the corresponding assignment tables (Fig. S5–S7 and Tables S3–S8).

**Fig. 4:**
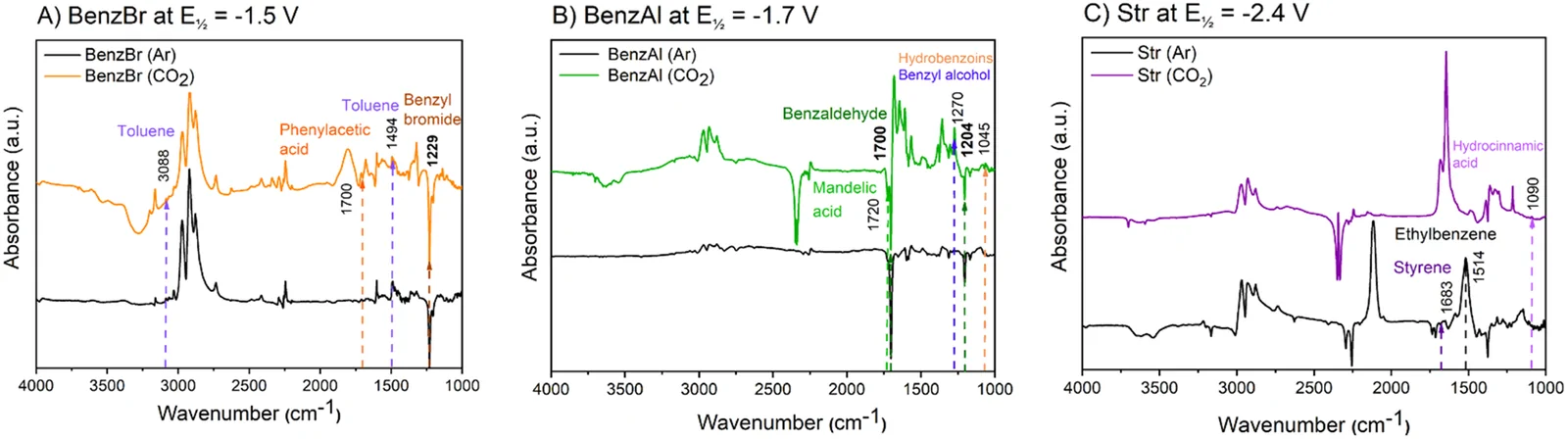
Electrochemical *in situ* FTIR spectra of the Cu_poly_ electrode in 0.1 M TBAPF_6_ acetonitrile electrolytes with 50 mM (A) benzyl bromide, (B) benzaldehyde, and (C) styrene at the halfwave potential of the CO_2_RR. Black spectra are measured in Ar-deaerated electrolytes whilst coloured spectra are in CO_2_-saturated electrolytes. For clarity we only highlight bands relating to substrate electroreduction. Background signals are recorded at −0.5 V *vs.* Ag/Ag^+^.

Fig. 4A shows common bands during the reduction of benzyl bromide in Ar-deaerated and CO_2_-saturated conditions. A clear and intense consumption band at 1229 cm^−1^ is observed in both conditions, which is assigned to the C–H wagging vibration of haloalkanes.^50^ In addition, bands at 3088 and 1494 cm^−1^ are assigned to the aromatic and alkane C–H stretching vibrations of the hydrogenation product toluene, respectively.^51^ In CO_2_-saturated electrolyte, an additional band is observed at 1700 cm^−1^ which is assigned to the C=O stretching vibration of the carboxylic acid group of phenylacetic acid.^52^ This assignment is consistent with the formation of phenylacetic acid observed during bulk electrolysis (Fig. 2A). It is important to note that the negative band related to the CO_2_ consumption at 2340 cm^−1^ is not observed in Fig. 4A. This observation is consistent with the interpretation that benzyl bromide activation is the main initial process under these conditions, followed by reaction of benzyl-derived intermediates with CO_2._ However, the absence of a strong 2340 cm^−1^ band does not by itself prove the sequence of elementary steps. Therefore, the FTIR data should be considered together with the CV and bulk electrolysis results, which collectively support a substrate-activated electrocarboxylation pathway for benzyl bromide.

Different features are observed in the case of benzaldehyde (Fig. 4B). In both Ar-deaerated and CO_2_-saturated conditions we observe the consumption of benzaldehyde at 1700 cm^−1^, assigned to the stretching vibration of the aldehyde C=O group.^53^ We also detect the in-plane stretching vibration of the phenyl ring C–H at 1204 cm^−1^, also related to benzaldehyde.^54^

Small bands related to alcohol C–O stretching vibrations of benzyl alcohol, the hydrogenation product, and hydrobenzoin, the dimerization product, are visible at 1270 cm^−1^ and 1045 cm^−1^, respectively.^52,55^ Interestingly, although these products do not arise from the electrocarboxylation, they are mainly identified in the presence of CO_2_, similar to the bulk electrolysis results (Fig. 2B). The exact reason for this is not clear. However, CO_2_ saturation may modify the local electrolyte environment and proton availability. The carbonate/bicarbonate-type bands observed in Fig. 4B indicate that CO_2_-derived species are present near the electrode surface, although their exact role in proton transfer cannot be determined from the present FTIR data alone. Such changes in the interfacial environment may contribute to the observed increase in hydrogenation products under CO_2_-saturation conditions.

The small positive band at 1720 cm^−1^ is assigned to the C=O stretching vibration of the carboxylic acid group of mandelic acid.^56^ In the case of BenzAl, a well-defined band at 2340 cm^−1^ is observed, indicating CO_2_ consumption. At the same applied potential, several small bands appear in the 1600–1300 cm^−1^ region, consistent with the formation of CO_2_ reduction products, as discussed above for Fig. 3B. These results agree with the bulk electrolysis discussion, where benzaldehyde reduction in the presence of CO_2_ leads mainly to dimerization and hydrogenation products, with only minor formation of the electrocarboxylation product.

Finally, Fig. 4C compares the spectra obtained during styrene reduction. Overall, the spectral response is dominated by bands associated with CO_2_ reduction products, while substrate-related features remain comparatively weak. In Ar-deaerated conditions, a small shoulder at 1514 cm^−1^ is observed and assigned to the C–C stretching vibration of ethylbenzene, indicating limited hydrogenation activity.^56,57^ In addition, a weak consumption band at 1683 cm^−1^, corresponding to the C=C stretching vibration of styrene, further confirms substrate reduction under these conditions.^58^

In CO_2_-saturated electrolyte, the spectra are largely dominated by bands associated with CO_2_ reduction products, particularly in the 1700–1200 cm^−1^ region. Only a very small band at 1090 cm^−1^, assigned to the C–O stretching vibration of hydrocinnamic acid, is observed.^58,59^ The low intensity of substrate-derived bands relative to the dominant CO_2_RR features is consistent with the bulk electrolysis results, where CO_2_-derived products account for the majority of the faradaic efficiency while electrocarboxylation products remain minor.

Taken together, the FTIR results suggest that styrene reduction and CO_2_ reduction occur simultaneously at highly cathodic potentials on Cu, with CO_2_RR becoming the dominant interfacial process under the applied conditions.

## 4. Conclusion

This work provided a framework for understanding electrocarboxylation on copper electrodes by linking substrate structure, applied potential, and competing electrochemical pathways. Across the three phenyl-activated substrates studied, electrocarboxylation selectivity is shown to arise from the interplay between substrate activation, CO_2_ reduction, and parallel side reactions, rather than from a single dominant mechanism.

Benzyl bromide exhibits the highest electrocarboxylation efficiency, which is consistent with facile substrate activation and subsequent reaction with CO_2_. In contrast, benzaldehyde forms a ketyl intermediate that predominantly undergoes dimerization and hydrogenation, limiting effective CO_2_ incorporation. For styrene, the more negative potentials required for substrate activation place the reaction in a region where CO_2_ reduction becomes increasingly competitive, limiting electrocarboxylation and favoring CO_2_-derived products.

Our results indicate that the relative positions of substrate activation and CO_2_ reduction potentials define distinct reactivity regimes, ranging from substrate-controlled behavior to conditions where CO_2_ reduction becomes increasingly important. Comparison with our previous studies on Pb cathodes further highlights the role of electrode material in controlling this balance. Whereas Pb favors substrate-controlled electrocarboxylation, the higher activity of Cu towards CO_2_ reduction introduces additional competing pathways that influence product selectivity.

These findings provide insight into how substrate structure, electrode material, and reaction conditions collectively govern electrocarboxylation outcomes and offer useful design principles for developing more selective electrochemical routes for CO_2_ utilization in organic synthesis.

## Conflicts of interest

The authors declare there is no conflict of interest.

## Supplementary Material

FD-OLF-D6FD00073H-s001

## Data Availability

All data supporting the findings of this study are available within the article. Supplementary information (SI): blank voltammogram of copper electrode in alkaline media, faradaic efficiencies of the bulk electrolysis of the three substrates in Ar, and the full potential resolved spectra for all investigated electrolyte environments, as well as the accompanying assignment tables. See DOI: https://doi.org/10.1039/d6fd00073h.
