# Supplementary material for: Competing pathways in electrocarboxylation on copper electrodes: defining reactivity regimes through substrate activation and CO2 reduction
Source: Faraday Discuss. 2026 Oct 9. Online ahead of print. doi: 10.1039/d6fd00073h (PMC13650555; doi:10.1039/d6fd00073h)
Supplement: Supplementary file 1 [file FD-OLF-D6FD00073H-s001.pdf]

Competing Pathways in Electrocarboxylation on Copper Electrode: Defining Reactivity Regimes through Substrate Activation and CO₂ Reduction

Eleni Menou Aikateriniadou[1,§], Connor Deacon-Price[1,§], Nina Chen[1], Myrthe Zwart[1,], Didjay F. Bruggeman[1,x,*], Amanda C. Garcia[1,*]

Van't Hoff Institute for Molecular Sciences, University of Amsterdam, Science Park 904, 1098 XH, Amsterdam, The Netherlands

(*) Corresponding author 1: a.c.garcia@uva.nl
Corresponding author 2: didjaybruggeman@gmail.com

(§) Authors have equally contributed the research.

.

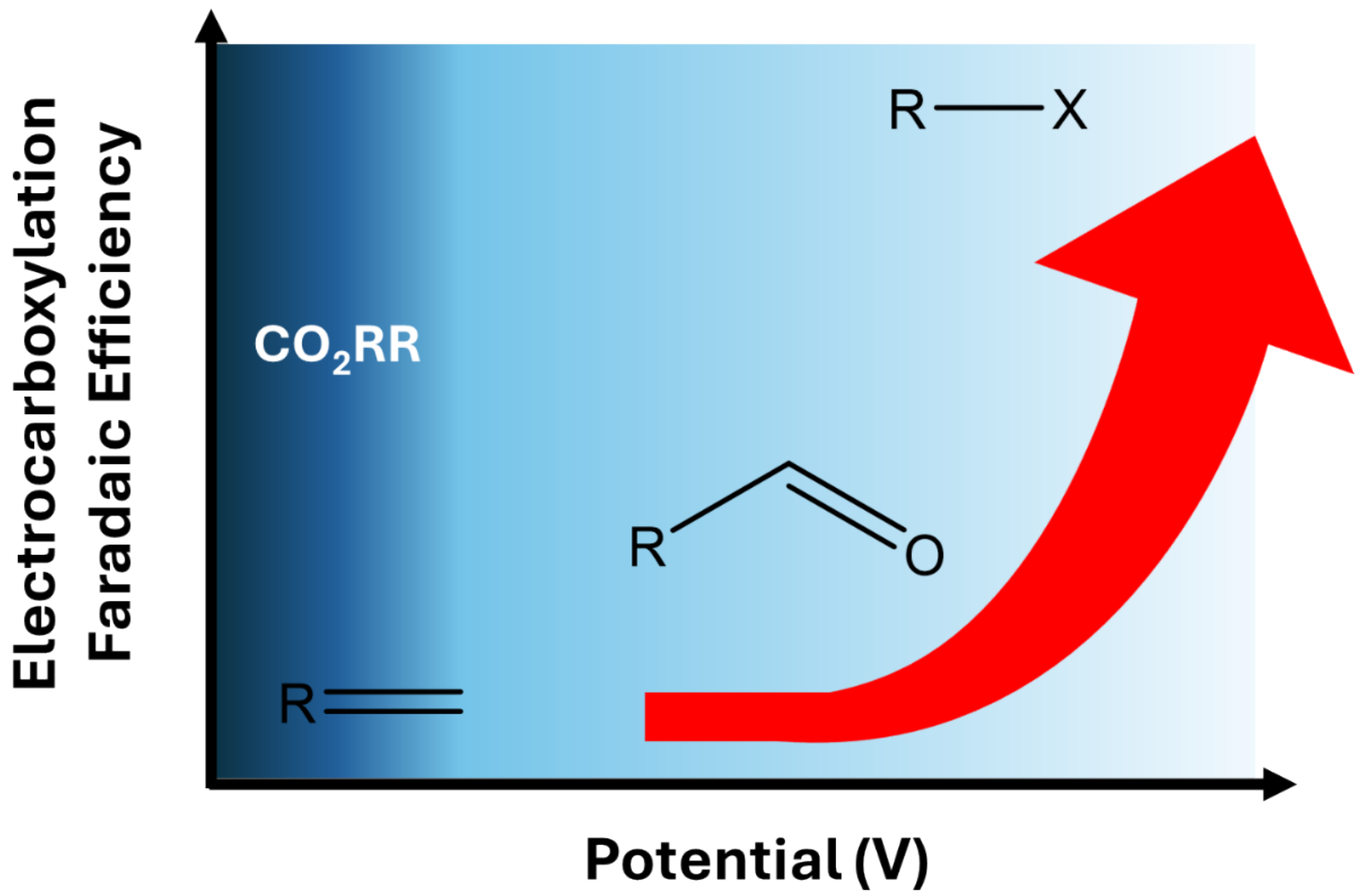

Table of Contents

Figure S1. Cyclic Votammogram of polycrystalline Cu electrode 3

Figure S2. Chronoamperometry experiments 4

Figure S3. Faradaic efficiencies of the substrates in the absence and presence of CO_2 5

Figure S4. Partial current densities of the substrates in the absence and presence of CO_2 5

Figure S5. Substracte conversion, yield and selectivity 6

Table S1. FTIR bands in Ar-dearated MeCN 6

Table S2. FTIR bands in CO_2-saturated MeCN 7

Figure S6. FTIR spectra of benzylbromide in absence and presence of CO_2 7

Table S3. FTIR bands in Ar-dearated benzylbromide 8

Table S4. FTIR bands in CO_2-saturated benzylbromide 8

Figure S7. FTIR spectra of benzaldehyde in absence and presence of CO_2 9

Table S5. FTIR bands in Ar-dearated benzaldehyde 9

Table S6. FTIR bands in CO_2-saturated benzaldehyde 9

Figure S8. FTIR spectra of styrene in absence and presence of CO_2 10

Table S7. FTIR bands in Ar-dearated styrene 10

Table S8. FTIR bands in CO_2-saturated styrene 11

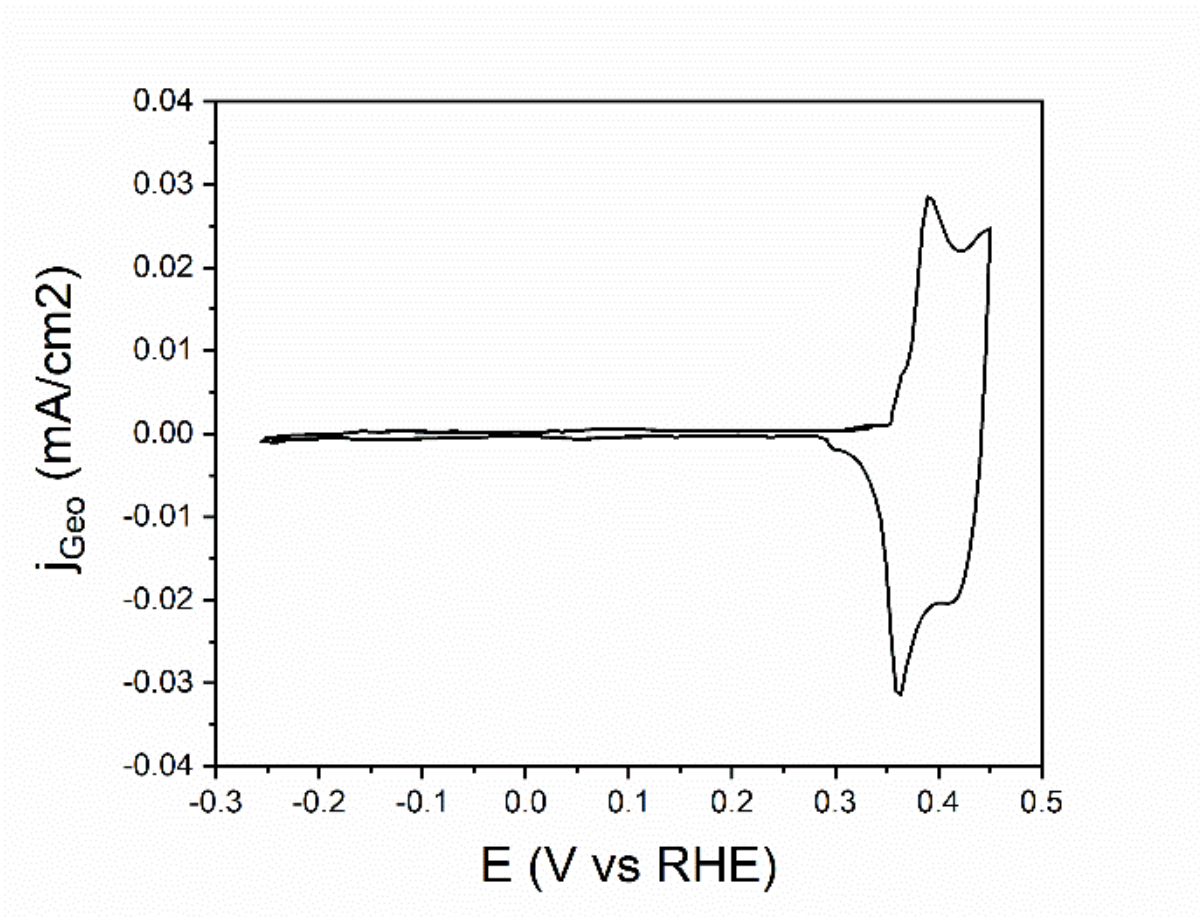

Figure S1. Cyclic voltammogram (CV) of polycrystalline Cu (Cu_{Poly}) in 0.1 M NaOH, under Ar atmosphere, scan rate at 50 mV/s from -0.25 V to 0.45 V vs. RHE. CE = Glassy Carbon; RE = RHE

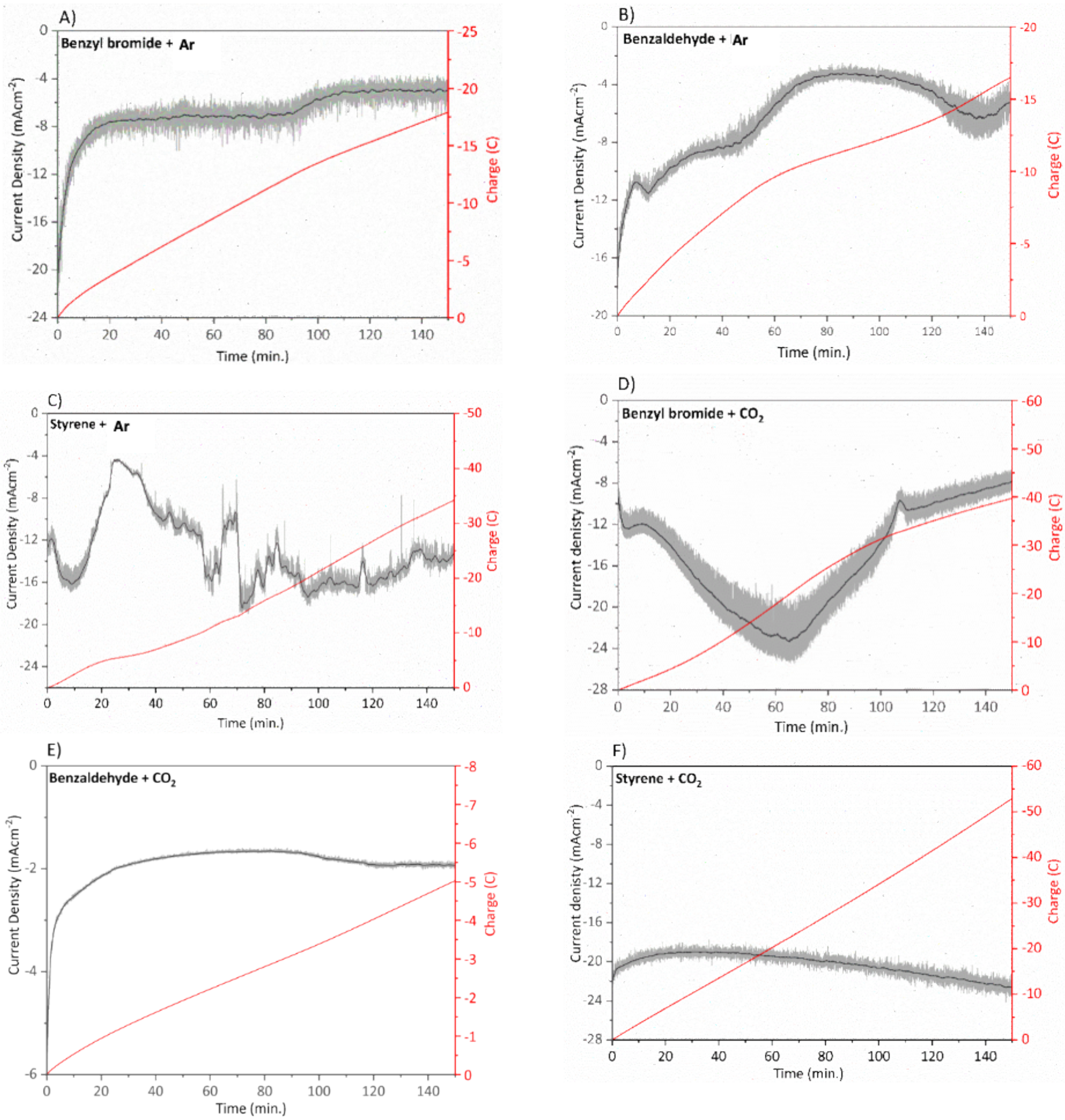

Figure S2. Chronoamperometry of Cu_{poly} electrode with Ar-deaearated 0.1 M $TBAPF_6$ MeCN electrolyte with 50 mM A) benzyl bromide, B) benzaldehyde, and C) styrene, and with CO_2-saturated 0.1 M $TBAPF_6$ MeCN electrolytes with 50 mM D) benzyl bromide, E) benzaldehyde, and F) styrene.

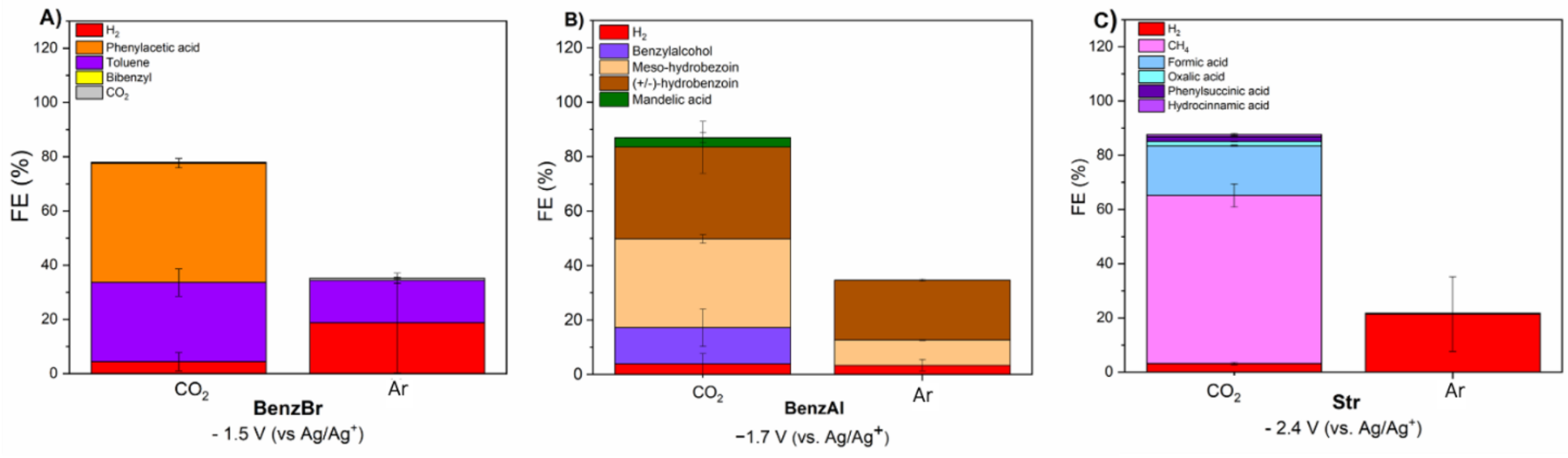

Figure S3. Faradaic efficiencies of the liquid and gas products quantified by HPLC and online GC using a Cu electrode and 50 mM of substrate; **A)** benzyl bromide, **B)** benzaldehyde or **C)** styrene in 0.1 M TBAPF$_6$ in CO_2-saturated (left column) and Ar-deaerared (right column) MeCN.

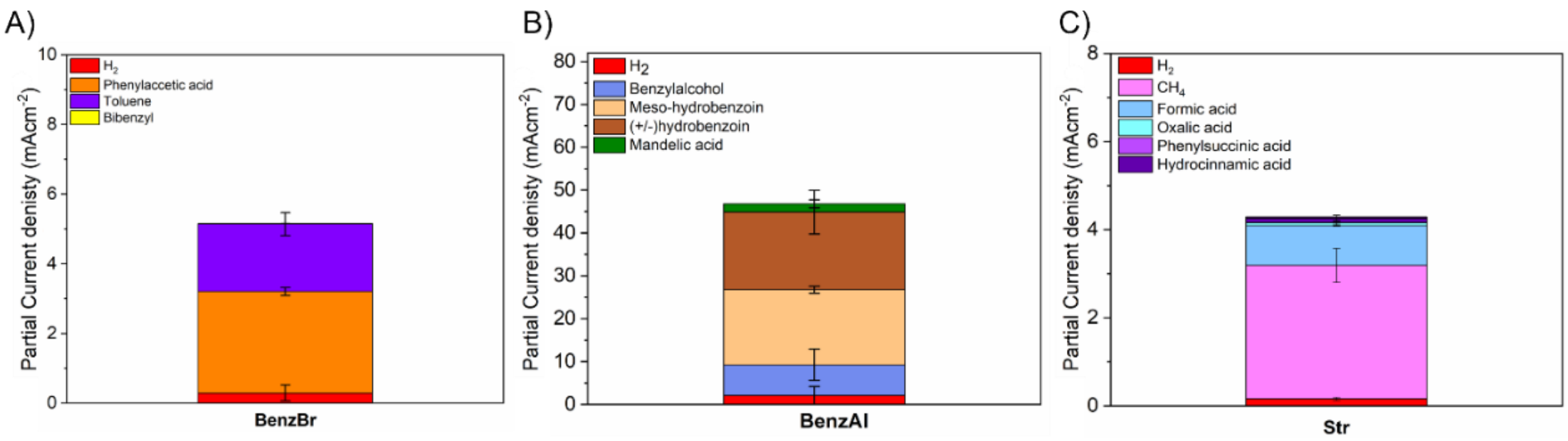

Figure S4. Partial current densities of the liquid and gas products quantified by HPLC and online GC using a Cu electrode and 50 mM of substrate; **A)** benzyl bromide, **B)** benzaldehyde or **C)** styrene in CO_2-saturated 0.1 M TBAPF$_6$. Values are calculated using data seen in Figures S2 and S3.

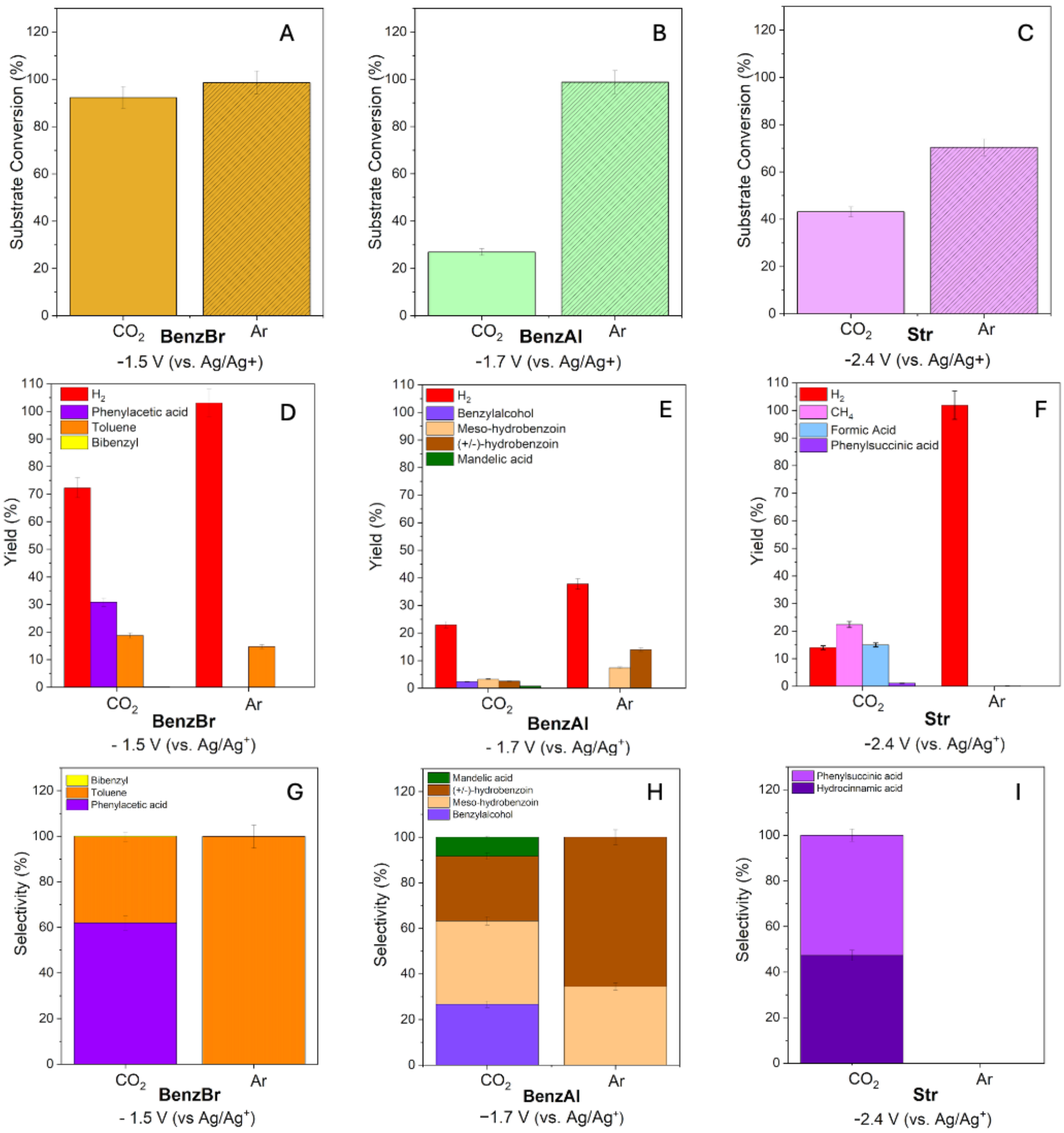

Figure S5. Substrate Conversion, Yiled and Selectivity towards carboxylated products of **A, D, G)** benzyl bromide, **B, E, H)** benzaldehyde and **C, F, I)** styrene, respectively, in Ar-deaerated and CO_2-saturated 0.1 M $TBAPF_6$.

Table S1. Assignment of the FTIR bands observed in dry and Ar deaerated MeCN electrolytes.[1, 2]

Wavenumber (cm^{-1})	Assignment	Compound
3640	ν O–H	H_2O
3544	ν O–H	H_2O
2975	ν C–H	TAA^+
2919	ν C–H	TAA^+
2877	ν C–H	TAA^+
2295	ν C–N	MeCN
2245	ν C–N	MeCN
2116	ν C□N	3-Aminocrotononitrile
1709	δ O–H	H_2O

1682	ν C=O	CH_3CONH_2
1528	ν C=N	3-Aminocrotononitrile

Table S2. Assignment of the FTIR bands observed in dry and CO_2 saturated MeCN electrolytes.[1,2]

Wavenumber (cm^{-1})	Assignment	Compound
3640	ν O–H	H_2O
3544	ν O–H	H_2O
2975	ν C–H	TAA^+
2919	ν C–H	TAA^+
2877	ν C–H	TAA^+
2340	ν C=O	CO_2
2245	ν C–N	MeCN
1681	ν C=O	CO_3^{2-}
1646	ν C=O	CO_3^{2-}
1607	ν C=O	HCO_3^-
1487	δ CH_3	CH_3CONH_2
1390	ν C–O	HCO_3^-
1362	ν C–O	CO_3^{2-}
1333	ν C=O	CO_3^{2-}
1305	ν C–O	CO_3^{2-}
1213	ν C–H	HCO_3^-

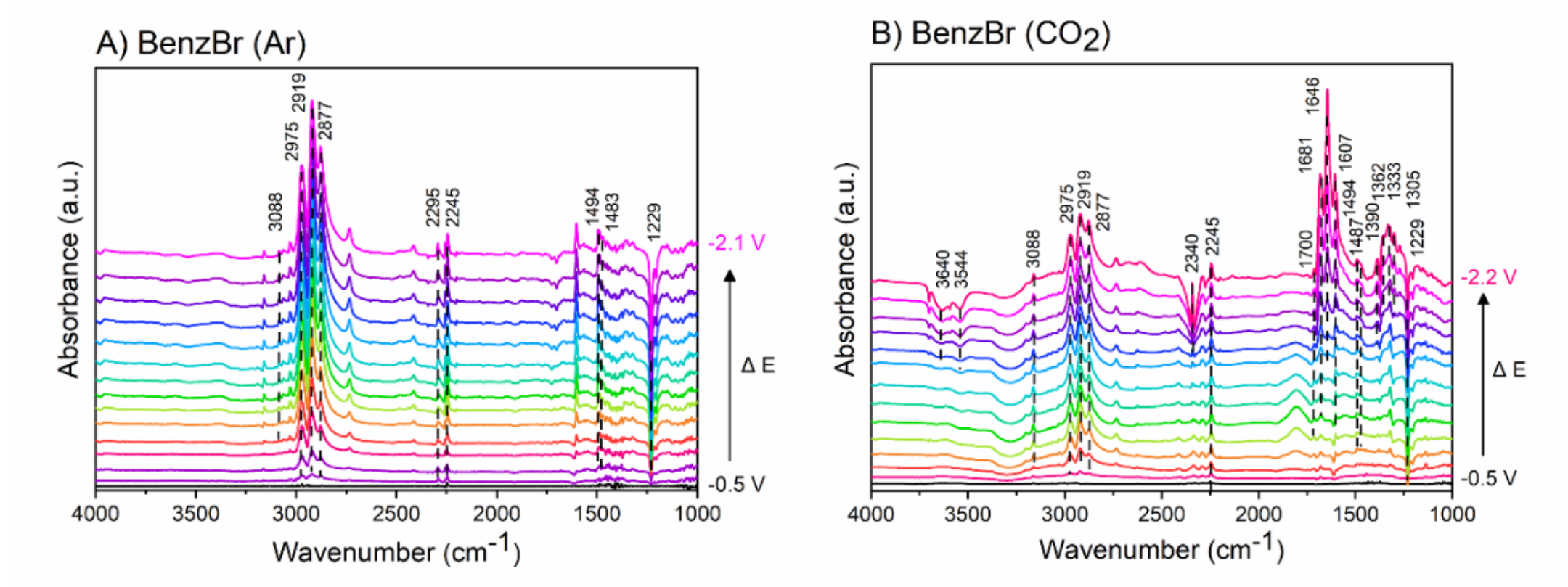

Figure S6. Electrochemical *in situ* FTIR spectra acetonitrile in 0.1 M $TBAPF_6$ electrolytes with 50 mM benzyl bromide in A) Ar-deaerated and B) CO_2-saturated conditions, at Cu_{Poly} WE. Background signals are recorded at -0.5 V vs Ag/Ag^+. Spectra in A) are recorded at -0.5 V, then -0.8 V, increasing by -100 mV until -2.1 V. Spectra in B) are recorded at -0.5 V, then -0.8 V, increasing by -200 mV until -1.2 V, increasing by -100 mV until -2.2 V.

Table S3. Assignment of the FTIR bands observed in dry and Ar deaerated benzylbromide containing MeCN electrolytes.[1-4]

Wavenumber (cm^{-1})	Assignment	Compound
3088	v C–H	Toluene
2975	v C–H	TAA^+
2919	v C–H	TAA^+
2877	v C–H	TAA^+
2295	v C–N	MeCN
2245	v C–N	MeCN
1494	v C–H	Toluene
1483	δ C–H	CH_3CONH_2
1229	ω C–H	Benzyl bromide

Table S4. Assignment of the FTIR bands observed in dry and CO_2 saturated benzylbromide containing MeCN electrolytes.[1-4]

Wavenumber (cm^{-1})	Assignment	Compound
3640	v O–H	H_2O
3544	v O–H	H_2O
3088	v C–H	Toluene
2975	v C–H	TAA^+
2919	v C–H	TAA^+
2877	v C–H	TAA^+
2340	v C=O	CO_2
2245	v C–N	MeCN
1700	v C=O	Phenylacetic acid
1681	v C=O	CO_3^{2-}
1646	v C=O	CO_3^{2-}
1607	v C=O	HCO_3^-
1494	v C–H	Toluene
1487	δ CH_3	CH_3CONH_2
1390	v C–O	HCO_3^-
1362	v C–O	CO_3^{2-}
1333	v C=O	CO_3^{2-}
1305	v C–O	CO_3^{2-}
1229	ω C–H	Benzyl bromide

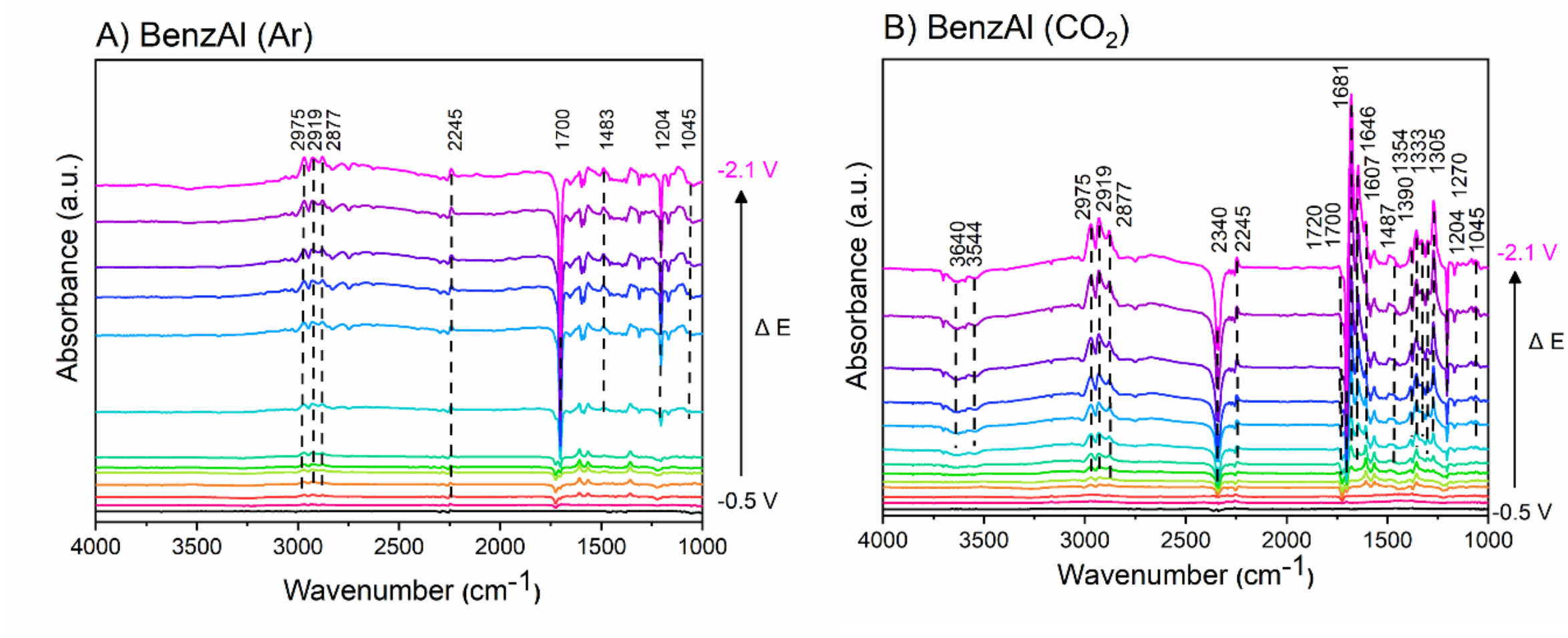

Figure S7. Electrochemical *in situ* FTIR spectra acetonitrile in 0.1 M $TBAPF_6$ electrolytes with 50 mM benzaldehyde in A) Ar-deaerated and B) CO_2-saturated conditions, at Cu_{Poly} WE. Background signals are recorded at -0.5 V vs Ag/Ag^+. Spectra in A) are recorded at -0.5 V, then -1.0 V, increasing by -200 mV until -1.4 V, then increasing by -100 mV until -2.1 V, then increasing by -200 mV until -2.5 V. Spectra in B) are recorded at -0.5 V, then -1.0 V, increasing by -200 mV until -1.4 V, increasing by 100 mV until -2.1 V, then increasing by -200 mV until -2.5 V.

Table S5. Assignment of the FTIR bands observed in wet and Ar deaerated MeCN benzaldehyde containing electrolytes.[1,2]

Wavenumber (cm^{-1})	Assignment	Compound
2975	v C–H	TAA^+
2919	v C–H	TAA^+
2877	v C–H	TAA^+
2245	v C–N	MeCN
1700	v C=O	Benzaldehyde
1483	δ CH_3	CH_3CONH_2
1204	v C–H	Benzaldehyde
1045	v C–O	Hydrobenzoins

Table S6. Assignment of the FTIR bands observed in wet and CO_2 saturated benzaldehyde containing MeCN electrolytes.[1,2,5,6]

Wavenumber (cm^{-1})	Assignment	Compound
3640	v O–H	H_2O
3544	v O–H	H_2O
2975	v C–H	TAA^+
2919	v C–H	TAA^+
2877	v C–H	TAA^+
2340	v C=O	CO_2
2245	v C–N	MeCN
1720	v C=O	Mandelic Acid

1700	v C=O	Benzaldehyde
1681	v C=O	CO_3^{2-}
1646	v C=O	CO_3^{2-}
1607	v C=O	HCO_3^-
1487	δ CH_3	CH_3CONH_2
1390	v C–O	HCO_3^-
1354	v C–O	CO_3^{2-}
1333	v C=O	CO_3^{2-}
1305	v C–O	CO_3^{2-}
1270	v C–O	Benzyl alcohol
1204	v C–O	Benzaldehyde
1045	v C–O	Hydrobenzoins

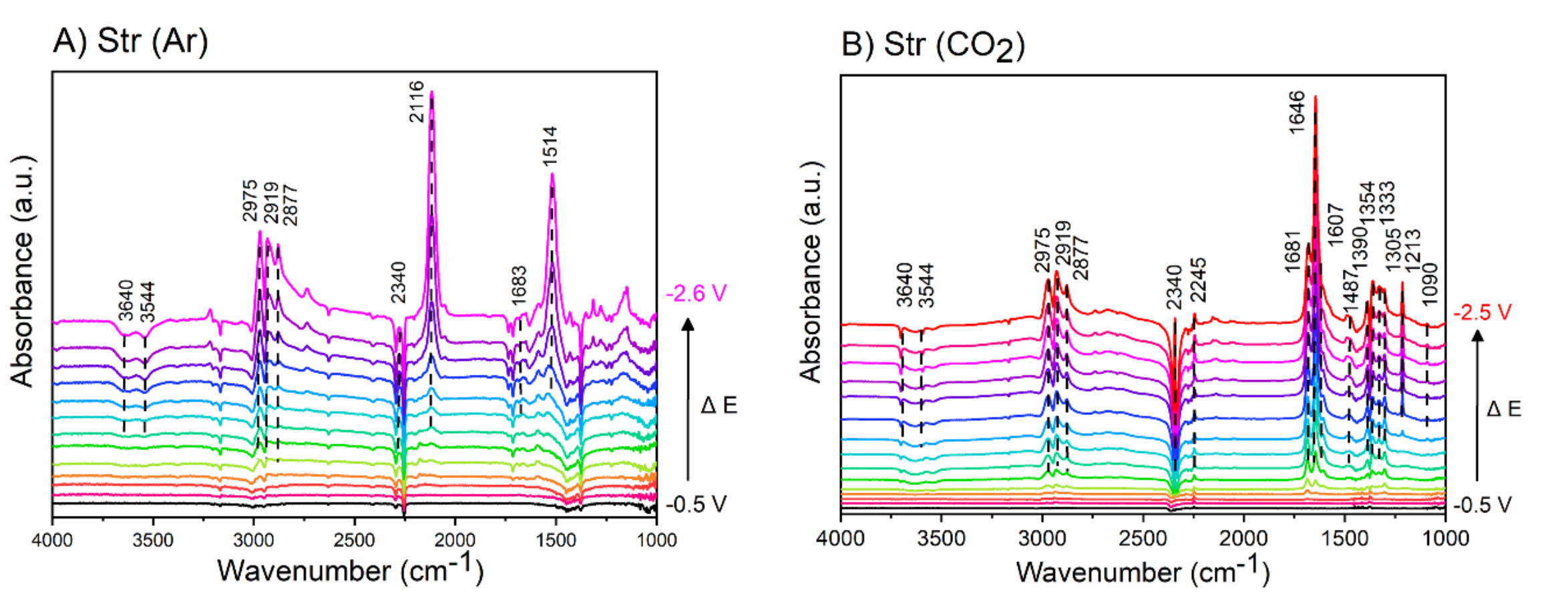

Figure S8. Electrochemical *in situ* FTIR spectra acetonitrile in 0.1 M $TBAPF_6$ electrolytes with 50 mM styrene in A) Ar-deaerated and B) CO_2-saturated conditions, at Cu_{Poly} WE. Background signals are recorded at -0.5 V vs Ag/Ag^+. Spectra in A) are recorded at -0.5 V, then -1.0 V, increasing by -200 mV until -2.0 V, then increasing by -100 mV until -2.6 V. Spectra in B) are recorded at -0.5 V, then -1.0 V, increasing by -200 mV until -1.4 V, increasing by 100 mV until -2.5.

Table S7. Assignment of the FTIR bands observed in wet and Ar deaerated MeCN electrolytes.[1,2,7]

**Wavenumber (cm^{-1})**	**Assignment**	**Compound**
3640	v O–H	H_2O
3544	v O–H	H_2O
2975	v C–H	TAA^+
2919	v C–H	TAA^+
2887	v C–H	TAA^+
2340	v C=O	CO_2
2116	v C≡N	3-Aminocrotononitrile
1683	v C=C	Styrene

1514	v C–C	Ethylbenzene

Table S8. Assignment of the FTIR bands observed in wet and CO_2 saturated MeCN electrolytes.[1,2,8]

Wavenumber (cm^{-1})	Assignment	Compound
3640	v O–H	H_2O
3544	v O–H	H_2O
2975	v C–H	TAA^+
2919	v C–H	TAA^+
2877	v C–H	TAA^+
2340	v C=O	CO_2
2245	v C–N	MeCN
1681	v C=O	CO_3^{2-}
1646	v C=O	CO_3^{2-}
1607	v C=O	HCO_3^-
1487	δ CH_3	CH_3CONH_2
1390	v C–O	HCO_3^-
1354	v C–O	CO_3^{2-}
1333	v C=O	CO_3^{2-}
1305	v C–O	CO_3^{2-}
1213	v C–O	Oxalate
1090	v C–O	Hydrocinnamic acid

References

1. Deacon-Price, C.; Changeur, L.; Santana, C. S.; Garcia, A. C. The Effect of the Tetraalkylammonium Cation in the Electrochemical CO2 Reduction Reaction on Copper Electrode. *ACS Catal* **2024**, 12928–12939. https://doi.org/10.1021/ACSCATAL.4C02297.

2. Foley, J. K.; Korzeniewski, C.; O N S, S. P. Anodic and Cathodic Reactions in Acetonitrile/Tetra-n-Butylammonium Tetrafluoroborate: An Electrochemical and Infrared Spectroelectrochemical Study. *Canadian Journal of Chemistry* **1998**, *66* (1), 201–206. https://doi.org/10.1139/V88-033.

3. Bertie, J. E.; Apelblat, Y.; Keefe, C. D. Infrared Intensities of Liquids XXV: Dielectric Constants, Molar Polarizabilities and Integrated Intensities of Liquid Toluene at 25 °C between 4800 and 400 Cm−1. *J Mol Struct* **2005**, *750* (1–3), 78–93. https://doi.org/10.1016/J.MOLSTRUC.2005.04.018.

4. Kawakami, K.; Tsuda, A. Brominated Methanes as Photoresponsive Molecular Storage of Elemental Br2. *Chem Asian J* **2012**, *7* (10), 2240–2252. https://doi.org/10.1002/ASIA.201200322.

5. Fujii, K.; Mitsudo, K.; Mandai, H.; Suga, S. Hydrogen Bonding-Assisted Enhancement of the Reaction Rate and Selectivity in the Kinetic Resolution of d,l-1,2-Diols with Chiral Nucleophilic Catalysts. *Adv Synth Catal* **2017**, *359* (16), 2778–2788. https://doi.org/10.1002/ADSC.201700057.

6. Lu, H.; Yan, Z.; Sun, M. Studies on Synthesis, Physicochemical Properties, Biological Activities of Two Novel Berberine-Based Salts with DL-Mandelate and Cinnamate Anions. *J Mol Struct* **2023**, *1294*, 136359. https://doi.org/10.1016/J.MOLSTRUC.2023.136359.

7. Linford-Wood, T. G.; Coles, N. T.; Webster, R. L. Room Temperature Iron Catalyzed Transfer Hydrogenation Using N-Butanol and Poly(Methylhydrosiloxane). *Green Chemistry* **2021**, *23* (7), 2703–2709. https://doi.org/10.1039/D0GC04175K.

8. Dürüst, Y.; Karakuş, H.; Kariuki, B. M.; Knight, D. W. Some Reactions of 2-(4-Substitutedphenyl)-2-(N-Methyl-N-4-Substitutedbenzamido) Acetic Acids. *Synth Commun* **2018**, *48* (17), 2206–2220. https://doi.org/10.1080/00397911.2018.1489966
